# Comparative growth rates of cultured marine dinoflagellates in the genus *Symbiodinium* and the effects of temperature and light

**DOI:** 10.1371/journal.pone.0187707

**Published:** 2017-11-29

**Authors:** Anke Klueter, Jennifer Trapani, Frederick I. Archer, Shelby E. McIlroy, Mary Alice Coffroth

**Affiliations:** 1 Department of Geology, State University of New York at Buffalo, Buffalo, New York, United States of America; 2 Department of Biological Sciences, State University of New York at Buffalo, Buffalo, New York, United States of America; 3 Southwest Fisheries Science Center, Marine Mammal and Turtle Division, NOAA Fisheries, La Jolla, California, United States of America; 4 Graduate Program in Evolution, Ecology and Behavior, State University of New York at Buffalo, Buffalo, New York, United States of America; University of Sydney, AUSTRALIA

## Abstract

Many dinoflagellate microalgae of the genus *Symbiodinium* form successful symbioses with a large group of metazoans and selected protists. Yet knowledge of growth kinetics of these endosymbionts and their ecological and evolutionary implications is limited. We used a Bayesian biphasic generalized logistic model to estimate key parameters of the growth of five strains of cultured *Symbiodinium*, *S*. *microadriaticum* (cp-type A194; strain 04–503), *S*. *microadriaticum* (cp-type A194; strain CassKB8), *S*. *minutum* (cp-type B184; strain Mf 1.05b.01.SCI.01), *S*. *psygmophilum* (cp-type B224; strain Mf 11.05b.01) and *S*. *trenchii* (cp-type D206; strain Mf 2.2b), grown in four different combinations of temperature and light. Growth kinetics varied among *Symbiodinium* strains and across treatments. Biphasic growth was especially evident for *S*. *minutum* and *S*. *psygmophilum* across all treatments. Monophasic growth was more common when final asymptotic densities were relatively low (~ 200 million cells ml^-1^). All species tended to grow faster and / or reached a higher asymptote at 26°C than at 18°C. The fastest growth was exhibited by *S*. *minutum*, with an approximate four-fold increase in estimated cell density after 60 days. The strongest effect of light was seen in *S*. *trenchii*, in which increasing light levels resulted in a decrease in initial growth rate, and an increase in asymptotic density, time when growth rate was at its maximum, final growth rate, and maximum growth rate. Results suggest that *Symbiodinium* species have different photokinetic and thermal optima, which may affect their growth-related nutritional physiology and allow them to modify their response to environmental changes.

## Introduction

Dinoflagellates (Pyrrhophyta; Dinophyceae) are a diverse group of unicellular protists best known for their formation of harmful algal blooms as well as for their symbiotic associations with cnidarian hosts such as corals, which demonstrates their great ecological as well as economic importance [1, 2]. Within a given habitat, dinoflagellate growth and population structure is driven by a combination of physical, chemical and biological factors [3]. Light energy, temperature, the availability of nutrients and CO_2_ and genotype are key factors that influence dinoflagellate growth patterns. In comparison to other microalgae, dinoflagellates have been shown to be relatively inefficient in the uptake of nutrients, demonstrated by slower growth rates overall [3]. They are however, extremely well adapted to specific niches [4] and can be found in a wide range of freshwater and marine environments [5].

Dinoflagellates of the genus *Symbiodinium* form symbioses with a variety of hosts including for example phyla such as foraminifera, ciliophora, and mollusca. They are, however, particularly well known for their role as endosymbionts within cnidarians where they play a key role in the construction of coral reefs. In such *Symbiodinium*-coral associations, the dinoflagellates reside within the host’s gastrodermal cells and the symbiont and host cells exchange organic and inorganic molecules that enable the growth and proliferation of both partners [6, 7]. The ecological importance of symbiotic dinoflagellates for the success of coral reef ecosystems has spurred the study of these dinoflagellates for more than three decades and continues with more urgency as coral reef health is threatened by increasing environmental pressures [8, 9]. Although our knowledge about these symbiotic partnerships increases rapidly, many features of the *Symbiodinium*-coral associations that are critical for reef health and survival remain poorly understood.

Genetic studies show that *Symbiodinium* is a highly diverse group of dinoflagellates that has been partitioned into nine major clades, A-I [10] and as molecular markers continue to evolve, phylotypes and species are being described [11–18]. The taxonomic diversity of *Symbiodinium* is reflected in functional differences, which in turn influences the phenotype of the entire symbiotic organism (cnidarian animal host and its associated eukaryotic and bacterial microbes). This is most often noticeable in the growth, reproduction and thermal tolerance of the coral [19–24]. A given *Symbiodinium* genotype found in a coral host can in part, determine the phenotype of that host and its susceptibility to environmental change. For example, the thermally tolerant D1 symbiont allows the coral host to increase its thermal tolerance by 1.5°C [25], yet this thermal tolerance comes at a cost for the coral. Reduced growth rates have been shown for corals that harbor D1 *Symbiodinium* as their dominant symbiont type [21, 26] due to their specific photokinetics, lipid production [26] and also to the amount of carbon translocated from the symbiont to the host [19]. Growth and survival of a reef-building coral is not only affected by the dominant *Symbiodinium* type harbored by the coral, but also how that genotype interacts with the environment. The coral *Pocillopora damicornis* harboring Clade D1 symbionts grows slower than their counterparts harboring Clade C1b-c symbionts, however, as temperatures increase coral growth is similar for both [27].

*Symbiodinium* genotypes have adapted to a wide range of spatial and temporal scales [28] and within those spatial and temporal scales, *Symbiodinium*-coral associations were found to be specific for a given host (e.g. [29–32]). Subsequently, many studies have addressed the effects of symbiont identity on the overall biological fitness of the symbiotic partnership. Hosts have been shown to change their symbiont composition in response to changes in environmental conditions [21, 25, 30, 33–36]. However, it is unclear exactly, how flexible hosts are to such changes [18, 37, 38]. Flexibility within the symbiont assemblage can allow for adaptation to environmental change and will depend on the physiology and growth kinetics of the composite *Symbiodinium* genotypes [39–41]. A study by Cunning *et al*. 2015 [42] suggests that symbiont populations within a host could be regulated in accordance with the costs and benefits to the symbiotic organism, which provides an additional perspective on the adaptability of this symbiotic partnership.

The population growth rate of a species is often dependent on an interaction between its intrinsic vital traits and the environment in which it is found. Indeed, recent studies have demonstrated how such information can significantly improve models that access coral bleaching predictability [43, 44]. Quantifying the growth rate of the population provides valuable information about its overall health and response to particular environmental conditions. To understand and evaluate growth kinetics as a proxy of biological fitness of *Symbiodinium* inside and outside of symbiosis and to better evaluate their ability to adapt to given environments, detailed information is needed on comparative growth rates of *Symbiodinium* species and how they are influenced by environmental factors.

To fill this gap and provide baseline information, we estimated several key parameters of the growth kinetics of five strains of *Symbiodinium* known to form viable symbioses with cnidarian hosts and often used in experimental studies. In order to better understand how environmental factors influence growth, cultures of each strain were grown in four combinations of temperature and light intensity. This modeling effort allows us to identify species-specific responses to temperature or light that may be relevant to survival and fitness. In addition, we aim to provide information to help define optimal growth conditions for *Symbiodinium* cultures, and further develop hypotheses for future studies examining physiological differences among strains, or the cellular basis of symbioses and the role that symbiont diversity plays.

## Materials and methods

### *Symbiodinium* identification and growth conditions

Cultures of five different strains of *Symbiodinium* were examined. For characterization of the symbiont taxa (here referred to as *Symbiodinium* or symbiont types / cp-type), a variable region in domain V of the chloroplast large subunit (23S) rDNA molecule was amplified as described in Santos *et al*. 2003 [45]. The five strains belonged to four different *Symbiodinium* species, *S*. *microadriaticum* (cp-type A194, ITS2 type A1, strain 04–503 and strain *Cass*KB8), *S*. *minutum* (cp-type B184, ITS2 type B1, strain Mf 1.05b.01.SCI.01), *S*. *psygmophilum* (cp-type B224, ITS2 type B2, strain Mf 11.05b.01) and *S*. *trenchii* (cp-type D206, ITS2 type D1a, strain Mf 2.2b). An overview of the *Symbiodinium* taxonomy and culture origin used in this study is provided in Table 1.

**Table 1:** *Symbiodinium* culture identification.

*Symbiodinium* ID	Cp–genotype	ITS2 –type	Culture ID	Culture since	Isolated from	Location	Reference
*S. microadriaticum*	A194	A1	*Cass* KB8	----	*Cassiopea* sp.	Hawaii, USA	[46, 47]
*S. microadriaticum*	A194	A1	04–503	2004	*Orbicella faveolata^a^*	Florida Keys, USA	[46, 47]
*S. minutum*	B184	B1	Mf 1.05b.01.SCI.01	2002	*Orbicella faveolata^a^*	Florida Keys, USA	[13]
*S. psygmophilum*	B224	B2	Mf 11.05b.01	2003	*Orbicella faveolata^a^*	Florida Keys, USA	[13]
*S. trenchii*	D206	D1a	Mf 2.2b	2002	*Orbicella faveolata^a^*	Florida Keys, USA	[13]

Cp-genotype, chloroplast genotype; ITS2-type, Internal Transcribed Spacer 2 genotype.

^*a*^*Symbiodinium* species is an incidental isolate that was most likely a surface contaminant as it is not a naturally occurring endosymbiont of *Orbicella faveolata*

Reference cultures were maintained in f/2 medium [48], 38 ppt salinity at 26°C under a 14:10 h 1ight:dark regime (70–90 *μ*mol photons m^-2^ s^-1^, from 40 W fluorescent lights) [49]. Samples of highly concentrated reference cultures were transferred into test tubes containing 10 ml of f/2 media. Experimental growth conditions were similar to conditions described in Klueter *et al*. 2015 [50]. Briefly, all *Symbiodinium* strains were maintained in four different combinations of temperature and light. Three samples of a homogenous culture of each *Symbiodinium* strain were maintained in f/2 media (*n* = 3 / treatment). The f/2 media had a pH of 7.70 that increased to 8.51 (± 0.2) (Fisher Scientific, pH Portable Meter, USA) when cultures were present. Experimental growth conditions are summarized in Table 2. Temperature was continuously monitored using the HOBO data logger system (Onset Computer Corporation, Bourne MA, USA). Light exposure followed a 14:10 h 1ight:dark regime. Light measurements were taken using a handheld light meter unit (LI-250A, Li-COR Inc., Biosciences, Lincoln, NE, USA), with a terrestrial radiation sensor (LI-190, Li-COR Inc., Biosciences, Lincoln, NE, USA). All test tubes were arranged randomly and rotated every two days to ensure all clones of all *Symbiodinium* strains experienced similar exposure to a given temperature and light treatment. In reporting results below, combined temperature and light treatments are identified in an abbreviated form using the codes listed in Table 2. For example, “T.18/L.049” refers to 18°C and 49 *μ*mol photons m^-2^ s^-1^.

**Table 2:** Experimental growth conditions.

Growth Condition	Temperature [°C]	Light [*μ*mol photons m^-2^ s^-1^]	Naming convention (this paper only)
1	18	48.6 (±4.4)	**T.18 / L.049**
2	26	48.6 (± 4.4)	**T.26 / L.049**
3	26	116.6 (± 6.01)	**T.26 / L.117**
4	26	230.6 (± 26.57)	**T.26 / L.231**

Cell densities of culture samples of all five *Symbiodinium* strains were determined using a hemocytometer (0.1 mm^3^, 0.1 mm depth; Neubauer Improved). Samples were harvested within two hours of the midpoint of the light cycle. To ensure an even distribution of algal cells within the f/2 media, samples were mixed thoroughly using a Pasteur pipette. When preparing culture samples for hemocytometer counts, care was taken that cell clumps were removed prior to counting. Densities of symbiotic dinoflagellates were calculated using four replicate counts for each aliquot.

For approximately the first week of the experiment, starting on Day 3, cells of symbiotic dinoflagellates were counted every day or in some cases every other day. During this period, a simple generalized logistic growth model was being developed and iteratively fit after every counting session in order to help determine when an asymptote had been reached and counting could cease. Approximately three weeks after the beginning of the experiment, it was determined that asymptotes had been reached in all treatments and counting should cease. However, further examination of poor fits in some of the single logistic curves suggested that the model should be modified to a biphasic form as well as to account for overdispersion. Thus counting was begun again at greater intervals to confirm the fit of the new model. During this period, around 40 days into the experiment, some treatments were inadvertently not counted for approximately one week, as models were being adjusted to test for attainment of asymptotic growth in different treatments. Counting concluded in all treatments 63 days after the beginning of the experiment. On average, there were 19 days on which cells were counted for each treatment (minimum = 16, maximum = 21).

### Modeling cell density

For each *Symbiodinium* strain grown at a given combination of temperature and light, we modeled cell growth using a Bayesian generalized logistic model. In order to account for heterogeneity of variance over time, we used an overdispersed Poisson likelihood model based on the related formulation of a negative binomial distribution [51]. We have formulated the model to simultaneously estimate growth parameters and quantify the uncertainty of monophasic or biphasic growth with a binary switching parameter, *w*, similar to the model averaging method described by Carlin and Chib 1995 [52].

The likelihood of observing a given cell density, *D* (x 10^4^ cells ml^-1^), *n* days after the experiment began was defined as:

D∼Poisson(μ∙ρ)

$$\mu =1+\frac{K_{1}-1}{1+e^{-B_{1}(n-M_{1})}}+w(\frac{k}{1+e^{-B_{2}(n-M_{1}-m)}})$$

ρ∼Gamma(α,α)

where (prior distributions in parentheses):

*μ* = Expected density,
*K*_*1*_ = Asymptotic density of the first curve (*Uniform*(1, 800)),
*k* = Increase in density of the asymptote of the second curve above *K*_*1*_ (*Uniform*(1, 500)),
*B*_*1*_, *B*_*2*_ = Logistic growth rates of the first and second curves respectively (*Uniform*(10^−1^, 1)),
*M*_*1*_ = Number of days to maximum growth rate of the first curve (*Uniform*(2, 40)),
*m* = Number of days to maximum growth rate of the second curve above *M*_1_ (*Uniform*(2, 40)),
*w* = A switching parameter determining if the model describes a monophasic logistic (= 0), or biphasic logistic (= 1) curve (*Bernoulli*(0.5)),
*ρ* = Gamma distributed error term,
*α* = Gamma shape and scale parameters (*Lognormal*(*μ =* 0, *τ* = 10^−4^)).

Posterior distributions were generated for each combination of the four treatments and five *Symbiodinium* strains (Tables 1 and 2) for a total of 20 curves using the *rjags* package in *R* v3.1.2 (R Core Team 2014). For the distributions, 10 independent MCMC chains were run, each with 1,000 adaptation steps, 1,000,000 steps for burn-in, and 1,000,000 sampling steps, thinned every 1,000 steps for a total of 10,000 samples from the posterior. Chain convergence and mixing were assessed with the Gelman-Rubin potential scale reduction factor (PSRF) as implemented in the R package *coda* [53].

In addition to estimating the parameters of the model, we also calculated the maximum asymptotic density (*K*_*max*_), which was *K*_*1*_ when *w* = 0, or *K*_*2*_ (= *K*_*1*_ + *k*) when *w* = 1. Maximum rate of growth (*R*_*max*_) across the entire curve was calculated as the first derivative of the likelihood function at *M*_*1*_ days when *w* = 0, and the maximum value of the first derivative at *M*_*1*_ or *M*_*2*_ (= *M*_*1*_ + *m*) days when *w* = 1. The time at maximum growth (*M*_*Rmax*_) was *M*_*1*_ when *w* = 0 or *B*_*1*_ > *B*_*2*_ when *w* = 1, otherwise it was *M*_*2*_ (when *B*_*2*_ > *B*_*1*_ and *w* = 1).

Statistical significance of differences between pairs of *Symbiodinium* strains and treatments for each parameter was assessed by subtracting the two distributions of interest and quantifying the proportion of the derived distribution that was greater than zero. Pairs with more than 97.5% or less than 2.5% of their distributions greater than zero were considered significantly different.

## Results

**Fig 1:**
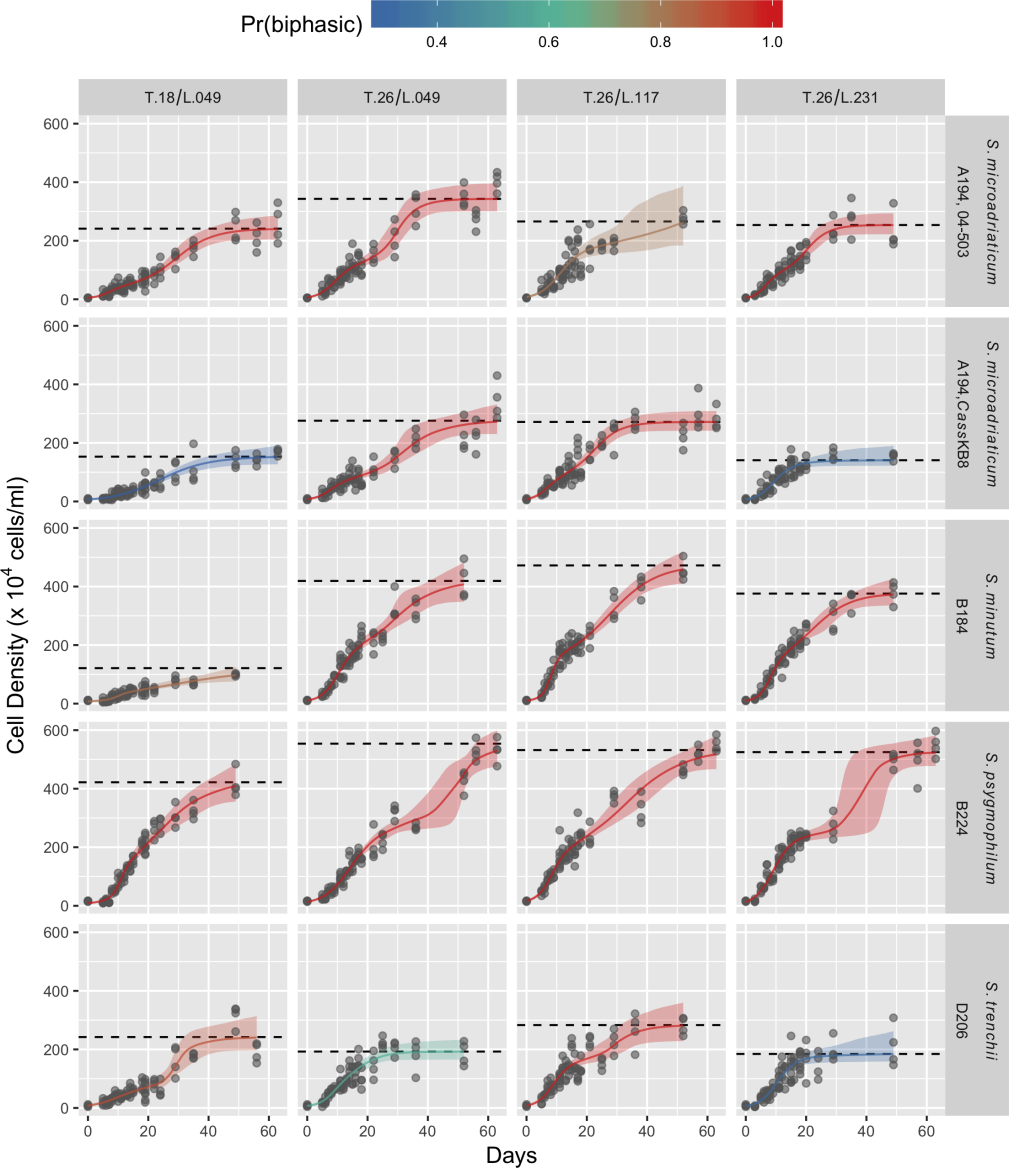
Growth of five different *Symbiodinium* strains (rows) in four combinations of temperature and light (columns). Grey points are actual cell counts. Colored lines and shaded areas are median estimated cell densities from Bayesian model and 95% credibility intervals. Colors denote the probability that the model is biphasic (Pr(biphasic)) from low (blue) to high (red) as estimated from the mean of the model switching parameter, *w*. Dashed grey lines are median estimates of maximum asymptotic density (*K*_*max*_).

The MCMC traces of samples from the posterior distributions showed good convergence and mixing with Gelman-Rubin potential scale reduction factor (PSRF) values being less than 1.08 for all parameters (S1 and S2 Figs, S1 Table). The estimated model fits to the data were good, with low uncertainty up until approximately day 20 in all curves (Fig 1).

The sparseness of count data after that led to an increase in uncertainty of the fit in many of the curves. Nonetheless, the fits demonstrate clear evidence of biphasic growth in most curves. This is supported by the posterior distribution of the model switching parameter, *w*, the mean of which across all posterior samples defines the probability that a biphasic model is a better fit than a monophasic model (Pr(biphasic), Table 3).

**Table 3:** Probability of biphasic growth in *Symbiodinium* exposed to different temperatures and light intensities.

	T.18 / L.049	T.26 / L.049	T.26 / L.117	T.26 / L.231
***S*.* microadriaticum*** A194 (04–503)	0.9926	0.9998	0.8126	0.9999
***S*.* microadriaticum*** A194 (*Cass*KB8)	0.2624	0.9993	0.9931	0.3273
***S*. *minutum*** B184	0.8334	0.9998	1	1
***S*. *psygmophilum*** B224	1	1	1	1
***S*.* trenchii*** D206	0.9093	0.5949	0.9913	0.3137

Probability of biphasic growth (Pr(biphasic) = mean of *w* switching parameter) for five *Symbiodinium* strains (rows) grown in four temperature and light treatments (columns). Treatments: Temperature, T [°C], Light, L [*μ*mol photons m^-2^ s^-1^].

The median Pr(biphasic) across all 20 curves was 0.996. Only three curves had a Pr(biphasic) less than 0.5: two treatments for *S*. *microadriaticum* (cp-type A194; strain *Cass*KB8) and one treatment for *S*. *trenchii* (cp-type D206; strain Mf 2.2b). It is notable that all three of these fits also had relatively low asymptotic cell densities. The minimum Pr(biphasic) was 0.262, while two treatments for *S*. *minutum* (cp-type B184; strain Mf 1.05b.01.SCI.01) and all treatments for *S*. *psygmophilum* (cp-type B224; strain Mf 11.05b.01) had Pr(biphasic) = 1.

There was considerable variability of parameter estimates across *Symbiodinium* strains as well as across experimental treatments of temperature and light, indicating that no two growth curves were exactly the same. Summaries of all parameters for each curve are presented in Table 4.

**Table 4:** Parameter posterior distribution from Bayesian double logistic models.

*Symbiodinium*	Treatment		Parameter posterior distribution from Bayesian double logistic models												
	T	L	*K*_*1*_	*k*	*K*_*2*_	*K*_*max*_	*B*_*1*_	*B*_*2*_	*M*_*1*_	*m*	*M*_*2*_	*R*_*M1*_	*R*_*M2*_	*R*_*max*_	*M*_*Rmax*_
***S*.* microadriaticum*** A194 (04–503)	**T.18**	**L.049**	36.55 (19.13–93.26)	202.99 (132.77–261.09)	241.54 (204.91–295.49)	241.3 (204.71–290.93)	0.45 (0.2–0.95)	0.16 (0.12–0.48)	7.26 (5.45–12.8)	21.89 (17.44–26.66)	29.31 (25.51–35.4)	5.15 (3.78–7.61)	8.2 (6.16–16.53)	8.22 (6.4–16.53)	29.11 (7.41–34.79)
***S*.* microadriaticum*** A194 (04–503)	**T.26**	**L.049**	119.1 (60.23–162.9)	226.27 (162.1–304)	343.12 (300.9–397.4)	343.09 (300.8–397.4)	0.35 (0.26–0.61)	0.27 (0.14–0.9)	9.15 (6.88–11.7)	20.27 (16.18–24.57)	29.64 (24.11–34.3)	10.53 (8.81–12.6)	15.12 (9.3–45.1)	15.13 (10.2–45.1)	29.33 (7.63–33.91)
***S*.* microadriaticum*** A194 (04–503)	**T.26**	**L.117**	175.54 (37.55–232.7)	132.53 (24.57–437)	293.15 (199.4–646)	265.86 (183.79–430)	0.29 (0.22–0.64)	0.44 (0.1–0.97)	11.1 (4.84–13.8)	24.92 (6.14–39.4)	36.17 (14.5–51.1)	12.68 (8.34–15.2)	10.2 (0.01–39.4)	13.66 (10.9–39.4)	12.73 (8.66–50.31)
***S*.* microadriaticum*** A194 (04–503)	**T.26**	**L.231**	80.28 (42.91–135)	174.4 (107.7–229.63)	253.72 (222.3–295)	253.72 (222.3–295)	0.52 (0.35–0.94)	0.26 (0.18–0.82)	6.54 (5.13–8.82)	13.35 (10.99–17.22)	20 (16.9–25.2)	11.87 (9.66–14.9)	11.68 (8.29–26.1)	12.9 (10.6–26.1)	17.08 (5.41–23.65)
***S*.* microadriaticum*** A194 (*Cass*KB8)	**T.18**	**L.049**	145.71 (13.11–181.2)	170.49 (9.05–481)	316.95 (140.4–634.2)	153.03 (127.3–209.8)	0.14 (0.11–0.75)	0.44 (0.04–0.97)	23.43 (9.01–27.7)	20.68 (3.13–39.17)	43.38 (25.37–63.6)	4.89 (3.23–5.96)	2.03 (0.1–11.24)	5 (4–11.24)	24.14 (17.4–57.34)
***S*.* microadriaticum*** A194 (*Cass*KB8)	**T.26**	**L.049**	68.68 (34.75–113.9)	210 (133.53–304.9)	275.89 (230.9–361.6)	275.85 (230.9–360.7)	0.37 (0.22–0.91)	0.14 (0.09–0.79)	7.95 (6.3–11.02)	23.91 (18.57–34.24)	31.99 (26.41–43)	7.21 (5.64–11.1)	7.87 (5.45–30.55)	9.07 (6.61–30.6)	29.03 (6.56–38.3)
***S*.* microadriaticum*** A194 (*Cass*KB8)	**T.26**	**L.117**	74.9 (32.5–151.4)	198.46 (114.24–257.34)	271.89 (241.84–311.78)	271.66 (241.71–309.15)	0.42 (0.25–0.96)	0.21 (0.15–0.78)	6.78 (4.9–10.63)	15.3 (12.16–19)	22.24 (18.53–27.7)	9.68 (7.43–13.5)	10.82 (7.7–24.9)	11.52 (9.12–24.9)	20.93 (5.58–26.7)
***S*.* microadriaticum*** A194 (*Cass*KB8)	**T.26**	**L.231**	133 (48.7–154.74)	133.23 (8.26–482)	264.77 (135.8–619.63)	140.83 (122.1–198.7)	0.34 (0.28–0.66)	0.45 (0.04–0.97)	9.35 (6.72–10.9)	18.55 (3.02–39)	27.8 (11.9–48.5)	11.18 (9.4–13.33)	0.74 (0–10.84)	11.22 (9.48–13.6)	9.41 (7–12)
***S*. *minutum*** B184	**T.18**	**L.049**	27.84 (15.2–99.1)	107.54 (30–431.4)	137.39 (85.6–518.9)	121.23 (77.24–220)	0.63 (0.13–0.98)	0.08 (0.05–0.9)	10.33 (8.49–18.7)	26.04 (6.34–39.4)	37.06 (19.9–53.6)	4.77 (2.88–6.95)	2 (0.06–7.47)	4.86 (2.95–8.04)	10.34 (8.51–48.72)
***S*. *minutum*** B184	**T.26**	**L.049**	175.36 (90.88–260.9)	261.3 (116.14–395.6)	419.2 (349.2–576.7)	419.14 (349.1–576.6)	0.36 (0.26–0.73)	0.14 (0.09–0.92)	10.42 (8.63–12.4)	19.21 (12.63–33.94)	29.59 (21.8–45.64)	17.81 (15.64–22)	9.89 (6.52–34.7)	18.31 (15.8–34.7)	10.47 (8.64–30)
***S*. *minutum*** B184	**T.26**	**L.117**	138.76 (115.1–166.6)	334 (277.4–412.45)	472.11 (415.2–558.5)	472.11 (415.2–558.5)	0.74 (0.55–0.95)	0.13 (0.11–0.16)	8.06 (7.57–8.67)	19.48 (15–25.73)	27.56 (22.92–34.1)	28.13 (23.3–33.5)	11.06 (9.1–13.23)	28.13 (23.3–33.5)	8.06 (7.57–8.67)
***S*. *minutum*** B184	**T.26**	**L.231**	127.77 (80.37–230.3)	250.6 (137.2–306)	375.91 (336.1–443.9)	375.91 (336.1–443.9)	0.58 (0.34–0.94)	0.16 (0.12–0.52)	8.14 (7.17–9.84)	13.92 (9.61–22.74)	22.04 (17.2–32.1)	21.73 (18.4–26.8)	10.34 (7.16–18.42)	21.84 (18.5–27.2)	8.14 (7.17–10.27)
***S*. *psygmophilum*** B224	**T.18**	**L.049**	112.08 (66.63–204.3)	314.18 (237.4–444.6)	421.98 (359.8–624.1)	421.98 (359.8–624.1)	0.73 (0.44–0.99)	0.15 (0.08–0.19)	10.9 (9.7–12.95)	14.23 (9.78–34)	25.05 (20–46.5)	24.4 (20.3–29.2)	11.41 (6.44–15.64)	24.4 (20.3–29.2)	10.9 (9.7–12.96)
***S*. *psygmophilum*** B224	**T.26**	**L.049**	268.77 (109.2–308.9)	295.56 (191–489.5)	553.85 (478.8–669.7)	553.85 (478.8–669.7)	0.22 (0.19–0.47)	0.26 (0.08–0.94)	14.6 (11.3–16.3)	34.63 (22.7–39)	49.3 (34.5–53.6)	14.78 (13.3–17.1)	17.41 (8.24–59.4)	17.88 (13.5–59.4)	45.15 (11.34–53.4)
***S*. *psygmophilum*** B224	**T.26**	**L.117**	168.48 (108–256.9)	372.05 (266.8–459)	531.94 (470.7–642.6)	531.94 (470.7–642.6)	0.43 (0.27–0.75)	0.11 (0.08–0.17)	8.39 (7.26–10.3)	24.48 (16.8–37.3)	32.93 (24.6–47)	20.06 (17–25.9)	10.24 (8.07–13.64)	20.09 (17–26.1)	8.39 (7.3–10.4)
***S*. *psygmophilum*** B224	**T.26**	**L.231**	240.68 (177.5–268.1)	283.3 (227.6–418.3)	524.97 (478.2–618.5)	524.97 (478.2–618.5)	0.32 (0.28–0.43)	0.44 (0.1–0.97)	9.18 (8.02–10.2)	29.14 (21.8–37.9)	38.28 (30.9–47.2)	19.2 (17.4–21.4)	29.99 (9.7–68.8)	30.01 (18–68.8)	33.85 (8.03–46.8)
***S*.* trenchii*** D206	**T.18**	**L.049**	75.7 (22.42–292.4)	164.1 (61.3–384.6)	244.35 (197.9–649.4)	242.22 (197.4–360)	0.23 (0.1–0.6)	0.53 (0.1–0.98)	10.23 (6.7–29.8)	19.59 (9.37–35.08)	29.77 (26.8–62.3)	4.5 (3.4–8.05)	18.88 (0.89–41.6)	18.88 (6.38–41.6)	29.37 (24.9–42.2)
***S*.* trenchii*** D206	**T.26**	**L.049**	141.36 (31.4–202.3)	147.97 (16.7–470.5)	215.72 (175.7–653.6)	192.68 (165.2–234.7)	0.32 (0.23–0.98)	0.25 (0.06–0.96)	10.24 (6.96–13.3)	11.58 (3.84–37.8)	21.17 (13.9–49.9)	12.5 (9.89–17.6)	6.29 (0–14.69)	12.65 (10.3–18)	11.08 (7.03–22.8)
***S*.* trenchii*** D206	**T.26**	**L.117**	160.19 (81.45–198.2)	129.43 (59.44–261.6)	283.81 (231.24–404.32)	283.16 (228.3–392.8)	0.37 (0.28–0.77)	0.3 (0.1–0.96)	8.92 (6.99–10.7)	20.45 (11–33.42)	29.45 (18.6–42.8)	14.76 (12.4–19.5)	9.27 (3.81–29.3)	15.43 (12.6–29.5)	9.21 (7–34.9)
***S*.* trenchii*** D206	**T.26**	**L.231**	175.09 (72.7–205.5)	159.31 (10.63–483.13)	329.14 (176.33–663.3)	184.48 (158.7–297.2)	0.33 (0.27–0.5)	0.48 (0.04–0.97)	10.44 (7.9–11.99)	21.78 (3.21–39.32)	32.17 (12.79–49.9)	14.36 (11.8–16.9)	0.82 (0–18.11)	14.46 (12.2–18.6)	10.53 (8.78–39.2)

Medians and 95% credibility intervals (parentheses) of parameter posterior distributions from Bayesian double logistic models. Treatments: Temperature, T [°C], Light, L [*μ*mol photons m^-2^ s^-1^]

Summaries and statistical significance of pairwise comparisons of parameters by species as well as temperature and light treatment are given in S2 and S3 Tables. Below we report results for the three biologically most important parameters derived from this model, maximum asymptotic density (*K*_*max*_), maximum rate of growth (*R*_*max*_), and time at maximum rate of growth (*M*_*Rmax*_).

**Fig 2:**
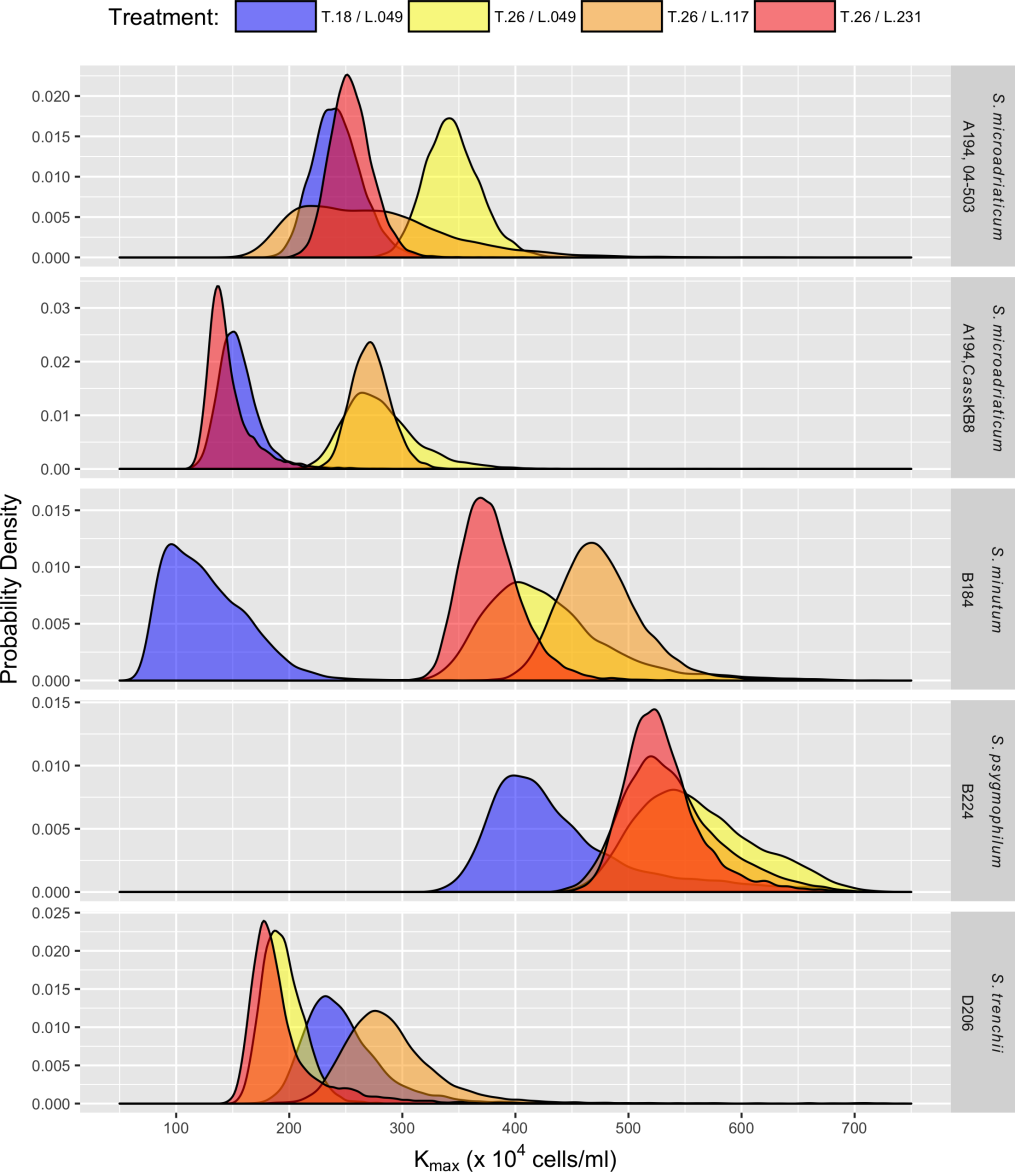
Maximum asymptote (*K*_*max*_). Posterior distributions of the maximum asymptote (*K*_*max*_) for five different *Symbiodinium* strains (rows) grown in four experimental temperature, T [°C] and light, L [*μ*mol photons m^-2^ s^-1^] treatments.

**Fig 3:**
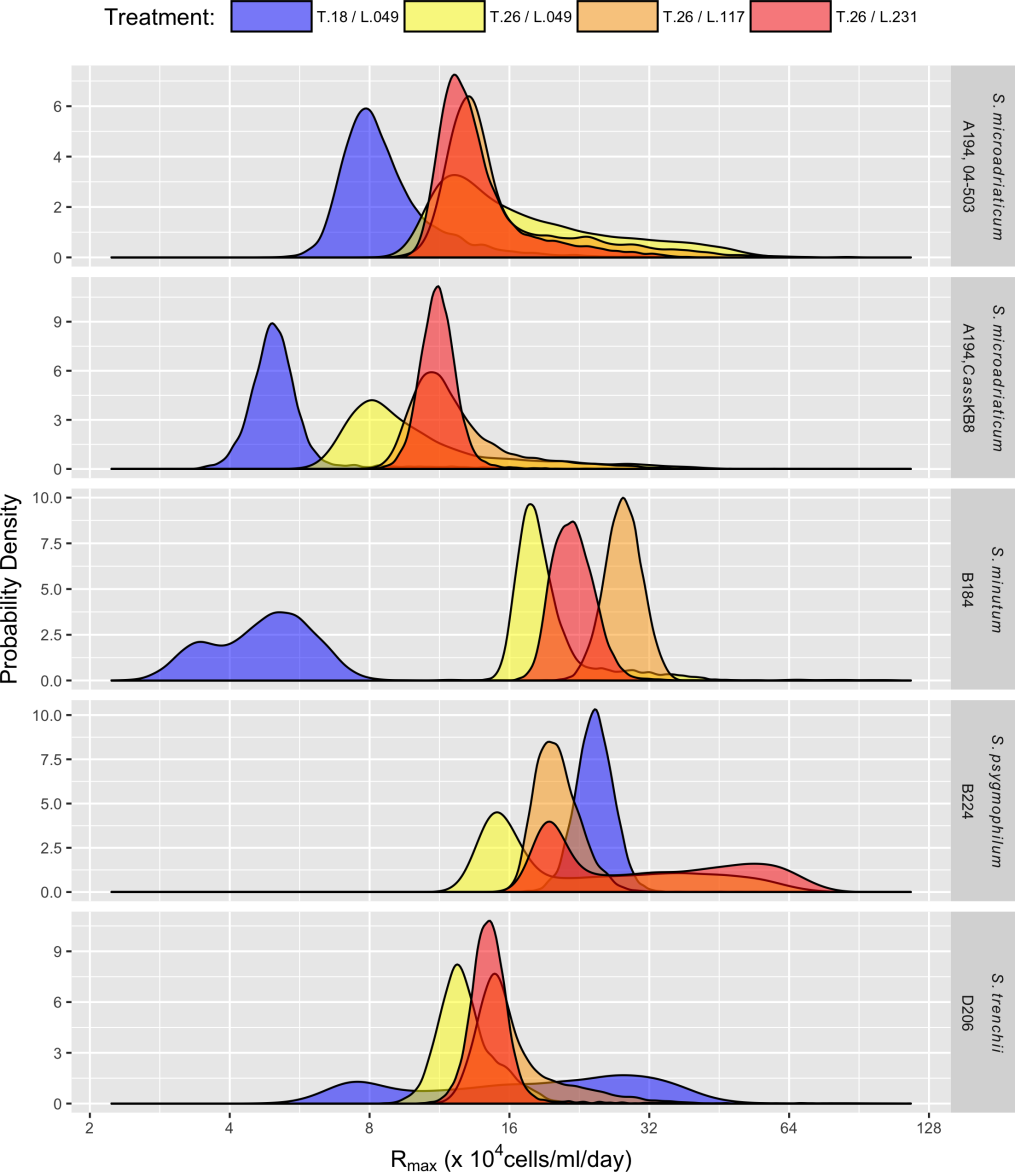
Maximum rate of growth (*R*_*max*_). Posterior distributions of the maximum rate of growth (*R*_*max*_) for five different *Symbiodinium* strains (rows) grown in four experimental temperature, T [°C] and light, L [*μ*mol photons m^-2^ s^-1^] treatments.

Both maximum asymptotic density (*K*_*max*_, Fig 2) and maximum growth rate (*R*_*max*_, Fig 3) were lowest for *S*. *minutum* (B184) grown at T.18/L.049 (121 x 10^4^ cells ml^-1^ and 4.9 x 10^4^ cells ml^-1^ day^-1^ respectively).

In contrast, *K*_*max*_ was greatest for *S*. *psygmophilum* (B224) grown at 26°C at all three light levels (~ 530 x 10^4^ cells ml^-1^). Median *R*_*max*_ was greatest for *S*. *psygmophilum* (B224) grown at T.26/L.231, but with a wide posterior reflecting a greater uncertainty stemming from a lack of data between approximately 30 and 50 days in this treatment. The modal *R*_*max*_ for this treatment was approximately 20 days, similar to the value seen in the same strain at T.26/L.117. The largest value of *R*_*max*_ with a relatively informative posterior was 28 x 10^4^ cells ml^-1^ day^-1^, seen for *S*. *minutum* (B184) grown at T.26/L.117.

**Fig 4:**
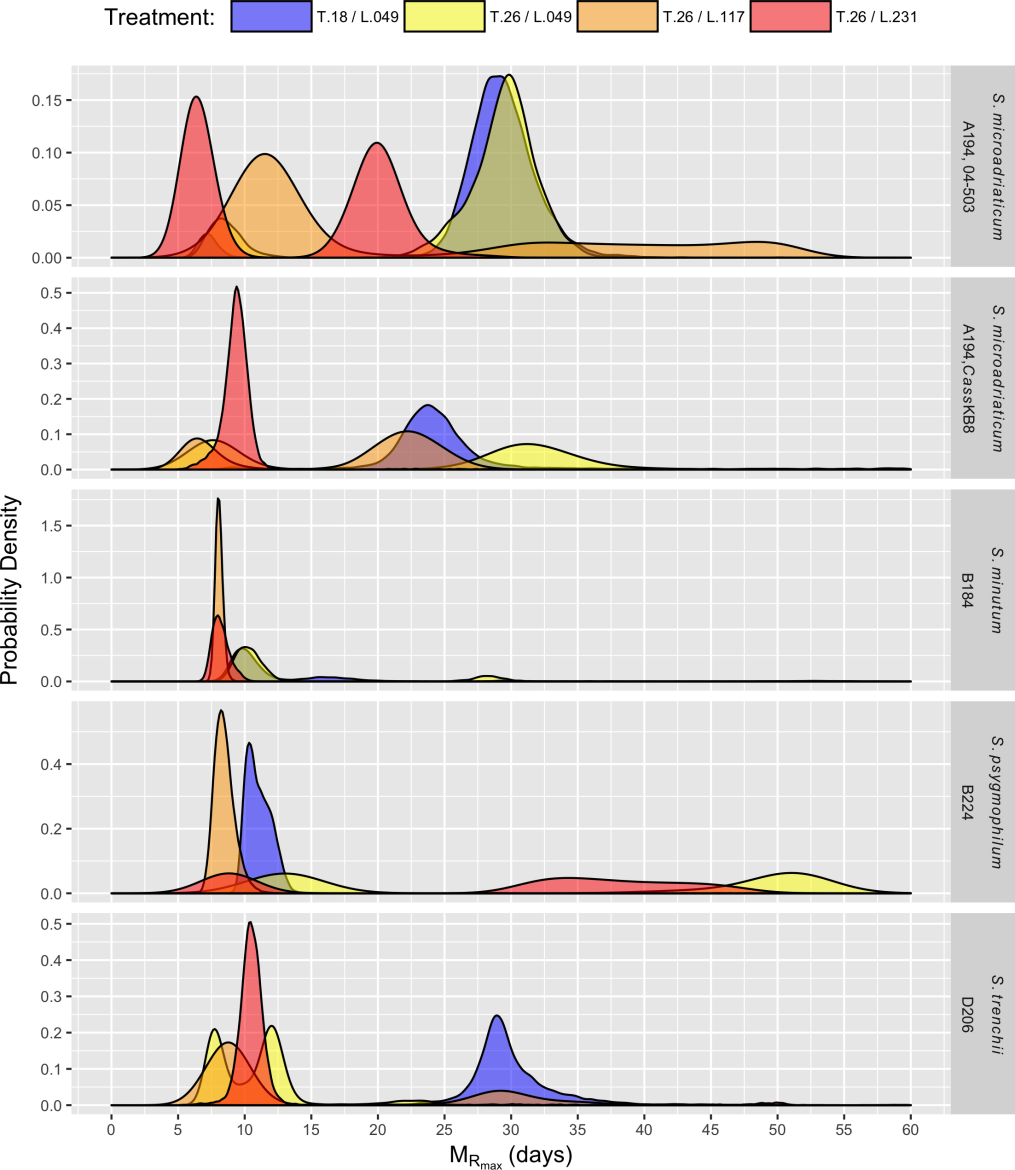
Time at maximum rate of growth (M_*Rmax*_). Posterior distributions of the time at maximum rate of growth (M_*Rmax*_) for five different *Symbiodinium* strains (rows) grown in four experimental temperature, T [°C] and light, L [*μ*mol photons m^-2^ s^-1^] treatments.

Maximum rate of growth was reached earliest by *S*. *minutum* (B184) grown at T.26/L.117 (*M*_*Rmax*_ = 8.1 days). Although the median value of *M*_*Rmax*_ is greatest for *S*. *psygmophilum* (B224) grown at T.26/L.049 (~ 45 days), the posterior distribution for this parameter is bimodal and very wide, reflecting uncertainty in this fit between approximately 40 and 50 days (Fig 4).

The largest estimates for *M*_*Rmax*_ with more informative posterior distributions are for *S*. *microadriaticum* (A194, strain 04–503) grown at T.18/L.049 and T.26/L.049 and *S*. *trenchii* (D206) grown at T.18/L.049, all reaching their maximum growth rates after approximately 29 days.

There were few consistent differences in parameters across treatments among the five *Symbiodinium* strains examined. However, *S*. *psygmophilum* (B224) had the greatest maximum asymptotic densities across all treatments (Table 3, Fig 2). Comparably, *S*. *minutum* (B184) also tended to have large values of *K*_*max*_ at 26°C, however this strain also had the lowest *K*_*max*_ for T.18/L.049. Overall, *S*. *trenchii* (D206) tended to have lower values of *K*_*max*_ across treatments. Because asymptotic density was large in *S*. *psygmophilum* (B224) and *S*. *minutum* (B184), these strains also tended to have the largest values of *R*_*max*_.

In all species but *S*. *trenchii*, (D206) maximum asymptotic growth was significantly greater at T.26/L.049 than at T.18/L.049 by approximately 100–300 x 10^4^ cells ml^-1^. In *S*. *trenchii* (D206), the increase from 18°C to 26°C at 49 *μ*mol photons m^-2^ s^-1^ was related to a decrease in *K*_*max*_ of 50 x 10^4^ cells ml^-1^. Maximum rate of growth (*R*_*max*_) was also significantly greater at 26°C than at 18°C in *S*. *microadriaticum* (A194, strain 04–503), *S*. *microadriaticum* (A194, strain *Cass*KB8), and *S*. *minutum* (B184). In *S*. *psygmophilum* (B224), the modal value of *R*_*max*_ was less at 26°C than at 18°C by approximately 5 x 10^4^ cells ml^-1^ day^-1^, but this difference was non-significant due to a broad posterior distribution for 18°C. Similarly, there was no difference in *R*_*max*_ between these two treatments for *S*. *trenchii* (D206) due to large uncertainty in the estimate at 18°C.

There was no consistent difference in *K*_*max*_ among the three light treatments at 26°C. However, in all *Symbiodinium* strains except *S*. *psygmophilum* (B224), where there was no significant difference among the three light treatments, *K*_*max*_ at 231 *μ*mol photons m^-2^ s^-1^ was significantly less than *K*_*max*_ at either 49 or 117 *μ*mol photons m^-2^ s^-1^. In all species except *S*. *microadriaticum* (A194, strain 04–503), *R*_*max*_ tended to be less at 49 *μ*mol photons m^-2^ s^-1^ than at 117 or 231 *μ*mol photons m^-2^ s^-1^, although in none of these comparisons was the difference significantly greater than zero.

As seen in Fig 4, several of the posterior distributions of the time at maximum growth rate (*M*_*Rmax*_) are bimodal. This likely reflects similar growth rates in the first and second parts of the biphasic curves as it is most evident in curves which at least visually do not appear to be strongly biphasic (e.g., *S*. *microadriaticum* (A194, strain 04–503) at T.26/L.231). Nonetheless, there are some similarities among treatments across species. For example, the primary modes of *S*. *microadriaticum* (A194, strain 04–503) and *S*. *trenchii* (D206) grown at 18°C both occur at approximately 28 days. Also, most species grown at 26°C have a mode where growth rate is at a maximum around 6–12 days. The second mode is more variable across *Symbiodinium* strains and treatments, occurring between 20 and 40 days in most species. As previously mentioned, the variability in this second mode is most likely the result of the lack of count data around this time in many treatments.

## Discussion

Effects of environmental conditions on dinoflagellate growth kinetics have been well described in a variety of studies ([54] and references within), often to better understand physical, chemical and biological mechanisms that lead to harmful algal blooms (e.g. [1, 55]) or to discover ideal growth conditions for bio-technological application [56]. On the other hand, a detailed understanding of growth kinetics in *Symbiodinium* species is still sparse. We investigated the growth of *S*. *microadriaticum* (A194; strain 04–503 and strain *Cass*KB8), *S*. *minutum* (B184; strain Mf 1.05b.01.SCI.01), *S*. *psygmophilum* (B224; strain Mf 11.05b.01) and *S*. *trenchii* (D206; strain Mf 2.2b), all of which were grown in four different combinations of temperature and light (Table 2). The results strongly demonstrated that growth kinetics varied among *Symbiodinium* types and across treatments. Also, for the first time, we were able to quantify biphasic growth for *Symbiodinium*, which was especially evident for *S*. *psygmophilum* (B224) and *S*. *minutum* (B184) across all treatments. Monophasic growth was more common when final asymptotic densities were comparatively low (~ 200 million cells ml^-1^). Clarifying growth kinetics of *Symbiodinium* will provide critical insight into the ecological diversity and adaptation capability of this very important group of dinoflagellates.

Ecologically vital characteristics of a species are often defined by quantifying the species growth rate. In algal cultures for example, it is a direct measure of inclusive fitness, and as such, a particularly informative trait to examine [57]. Parameters such as temperature and light intensity are known to directly influence algal population growth and sophisticated models that help improve the understanding of how these factors affect growth kinetics are emerging [43, 58, 59]. Biphasic relationships can be found throughout natural systems in particular those related to aspects of nutrition [60]. Here biphasic models have been shown to be particularly well suited for describing the inflection points observed during nutritional changes [60].

Although to our knowledge biphasic models have not previously been applied to growth profiles in *Symbiodinium*, we found that they were good fits to our data and useful for comparing the effects of different environmental conditions on the growth of individual *Symbiodinium* species. For example, strong biphasic responses were particularly evident for the *Symbiodinium* species *S*. *psygmophilum* (B224) and *S*. *minutum* (B184). For both species, biphasic growth was observed across all temperature and light treatments, but it was especially strong in 26°C at low light intensity (49 *μ*mol photons m^-2^ s^-1^) and for *S*. *psygmophilum* (B224) also at highest light intensity (231 *μ*mol photons m^-2^ s^-1^). Although both species fall within Clade B, *S*. *minutum* (B184) and *S*. *psygmophilum* (B224) are known to differ in their physiology and ecology [13, 50]. Results from our study show that these differences are also expressed by different growth kinetics.

Apart from examining different *Symbiodinium* species, we also examined two strains of a single *Symbiodinium* species belonging to Clade A, *S*. *microadriaticum* (A194, strain 04–503 and *Cass*KB8). Similar to *S*. *psygmophilum* and *S*. *minutum* biphasic growth in both strains of *S*. *microadriaticum* was also most evident in 26°C at 49 *μ*mol photons m^-2^ s^-1^, but at the same time, clear differences in growth kinetics between the two *S*. *microadriaticum* strains were evident. The probability of biphasic growth in *S*. *microadriaticum* (A194; *Cass*KB8) was significantly lower when exposed to 18°C and 49 *μ*mol photons m^-2^ s^-1^ as well as 26°C and 231 *μ*mol photons m^-2^ s^-1^. This may indicate narrower growth optima at conditions between these two treatments. On the other hand, *S*. *microadriaticum* (A194; 04–503) showed biphasic growth in all four temperature and light combinations. In *S*. *trenchii* (D206) however, biphasic growth was more prevalent at 18°C and 49 *μ*mol photons m^-2^ s^-1^and in 26°C at 117 *μ*mol photons m^-2^ s^-1^. Overall a more monophasic growth pattern was only observed in four instances and only for two *Symbiodinium* strains (*S*. *microadriaticum* (A194; *Cass*KB8) at 18°C and 49 *μ*mol photons m^-2^ s^-1^ and 26°C at 231 *μ*mol photons m^-2^ s^-1^, and *S*. *trenchii* (D206) at 26°C and 49 and 231 *μ*mol photons m^-2^ s^-1^). Interestingly, different biphasic growth patterns were also found in a recent study by Karim *et al*. 2015 [41]. The authors investigated the effects of three different temperatures (25°C, 30°C and 33°C) on selected *Symbiodinium* species belonging to Clades A, B, C, D and F and noted biphasic growth patterns in *Symbiodinium* cultures exposed to higher temperatures. Although growth was not specifically modeled in this study, results by Karim *et al*. highlight the importance of more quantitatively examining growth to better understand the cell cycle of *Symbiodinium* and how it is affected by physiological and ecological differences. It is important to note, that near the end of our experiment, cell counts were taken farther apart in time, due to experimental conditions. This resulted in a gap between counts of as much as 20 days. There was also variability in the attainment of asymptotic growth among treatments. Both of these issues result in greater uncertainty of many of the parameter estimates for the upper curves relative to the lower. Finally, all recognizable cells were counted without distinguishing between living and dead cells. Thus, care should be taken in interpreting the results of these parameter estimates in more than a relative sense. This being said, our analyses show that the general patterns reported here are expected to hold with a longer time series of counts, but more precision is likely and perhaps even higher final asymptotes, especially for *Symbiodinium* cultures still demonstrating growth at the end of this experiment.

As we exclusively monitored the effects of temperature and light on the growth of our cultures, we are only able to speculate as to some of the influential factors of the biphasic growth kinetics observed in this study. It has been firmly established that microbial interactions play an important role in the dynamic of phytoplankton populations and nutrient cycling, where they are tightly linked to the availability of organic carbon and inorganic nutrients ([61] and references within). Moreover, Bolch *et al*. (2017) [62] demonstrate that associated microbial interactions are likely to be equally important for dinoflagellate growth patterns as for example temperature and light. As *Symbiodinium* grow poorly in the absence of bacteria [63, 64], culturing media used in this study also contained bacteria communities, but of unknown composition. Culturing media was the same for all samples, but differences among *Symbiodinium* species in exponential growth, stationary and death phases are likely to affect the bacterial community within a sample and therefore nutrient availability and carbon cycling. Additionally, findings by Jeong *et al*. [65] demonstrated heterotrophic feeding strategies for a cultured free-living *Symbiodinium* species, *S*. *voratum* (Clade E1) [11]. The authors showed that *S*. *voratum* was able to ingest bacteria as well as small algal species. A heterotrophic feeding strategy would be of particular value when nitrogen and phosphate are depleted, which would cause autotrophic growth of *Symbiodinium* to slow down and eventually cease completely. A variety of symbiotic dinoflagellates are known to exist in a motile free-living state, although their physiology is not as well studied as their endosymbiotic counterparts [66, 67] Given that *Symbiodinium* species used in this study have been in culture for 10–15 years, it is possible that they can also employ feeding strategies of mixotrophs and that the biphasic growth kinetics reflect a shift in nutritional strategies (from autotrophic to heterotrophic feeding behavior).

Another aspect to consider is that the life cycles of *Symbiodinium* are more plastic than previously believed; the occurrence of meiosis might be more frequent than previously recognized. This can be seen in the dinoflagellate *Alexandrium minutum*, which undergoes cell divisions in both, diploid and haploid phases [68]. This finding calls for a more detailed look into cell cycle transitions in *Symbiodinium*.

In symbiosis, *Symbiodinium* species are recognized for their biological and physiological differences and their ability to adapt to a wide range of specific niches, ranging from different photosynthetic membranes to preferences for specific substrates and temperatures optima, to name just a few (e.g. [67, 69–71]). In fact, their symbiotic associations greatly depend on such niche adaptation (e.g. [72–75]). The results of our study further highlight the diversity of *Symbiodinium* species, each of which demonstrated distinct patterns of population growth. Greatest growth across all treatments was achieved by *S*. *psygmophilum* (B224), a species predominantly found in temperate waters [76], yet it is know to also grow in tropical waters [13, 77]. The final asymptotic density for this *Symbiodinium* species was significantly greater than that reached in any other species, indicating physiological processes that are suited to generating large populations in a variety of environmental conditions. Due to experimental limitations, only a few moderate temperature and light changes could be tested. Treatments in this study were similar to temperature and light intensities often experienced in temperate environments and they were also similar to the temperature and light intensity that the *Symbiodinium* cultures have been exposed to for the last 10–15 years. Hence findings of this study do not allow assumptions as to how the five *Symbiodinium* strains would respond to temperature and light conditions that are known to disrupt and / or damage the photosynthetic apparatus. However, our findings do provide useful baseline information that can aid in the informed design of studies examining growth patterns of *Symbiodinium* under more extreme environmental conditions.

*Symbiodinium* species have been shown to operate along a wide variety of physiological optima along a continuum from generalists to specialists [78]. The functional performance of a symbiotic association greatly depends on the genetic identity of its endosymbionts and its energetic success is likely to be driven by variations in the photokinetics of individual *Symbiodinium* species (e.g. [69, 79–81]). For instance, some *Symbiodinium* species belonging to Clade D appear to be particularly well adapted to high temperature stress and thus provide their symbiotic partner with a greater thermal tolerance [25, 38, 82]. A reduced rate of electron transport and capacity to absorb light appear to allow for an increased thermal tolerance in these Clade D *Symbiodinium* [83]. In this study, growth of the Clade D *Symbiodinium S*. *trenchii* (D206), was modest with highest density estimates of 284 x 10^4^ cells ml^-1^ at 26°C and 117 *μ*mol photons m^-2^ s^-1^. At the two higher light intensities (117 and 231 *μ*mol photons m^-2^ s^-1^) at 26°C, patterns of growth in *S*. *trenchii* (D206) were more similar to those observed for *S*. *microadriaticum* (A194; *Cass*KB8) than they were for any of the other three *Symbiodinium* strains. Both *Symbiodinium* species, *S*. *trenchii* (D206) and *S*. *microadriaticum* (A194; *Cass*KB8) showed monophasic growth at the higher temperature and light treatment (26°C and 231 *μ*mol photons m^-2^ s^-1^) indicating that an increase in light intensity cannot stimulate growth further.

Photosynthetic efficiency of *Symbiodinium* was not monitored in this study, but as this example highlights, it will be beneficial to investigate growth parameters in concert with photo-physiological measures. It is noteworthy that similarities between *S*. *trenchii* (D206) and *S*. *microadriaticum* (A194; *Cass*KB8) were also shown with regard to their metabolite profiles. When these species were grown in 18°C and 26°C, metabolites like inositol, C29 sterols and selected fatty acids were expressed in similar amounts, indicating potential similarities in their physiological make-up and performance [50]. Interestingly, in *S*. *microadriaticum* (A194), growth itself was about similar between the two strains 04–503 and *Cass*KB8 (Fig 3), however the estimated asymptotic density for strain 04–503 was significantly greater than it was for strain *Cass*KB8 (Fig 1). This was true for three out of the four treatments. Although genetically, both *Symbiodinium* strains are considered to be *S*. *microadriaticum* (A194), it is possible that a more detailed genetic analysis will assign the two strains to different taxa, explaining differences in their physiology as exhibited by the different growth kinetics observed in our study. It should also be mentioned that *Symbiodinium* used in this study have been in culture for a long time, at 26°C and 70–90 *μ*mol photons m^-2^ s^-1^, therefore adaptation to culture conditions is likely and needs to be evaluated critically when comparisons are made to their wild-types.

Results of this study demonstrate that growth kinetics vary among *Symbiodinium* strains and also with changing temperature and light intensity. Growth parameters such as *K*_*ma*x_ and *R*_*max*_ allow us to investigate species-specific responses to temperature and light. Such species-specific responses are likely to be vital for the survival and fitness of these species and consequently *Symbiodinium* growth rate in general [24]. Given our findings, we hope to motivate future research examining the diverse physiological variations in *Symbiodinium* spp. that drive population growth. Here factors such as temperature, light, CO_2_, nutrients, salinity, pH and microbial associations are of particular interest. Significant strides have already been made, for example, by investigating photo-physiological differences in CO_2_-concentrating mechanisms [54, 84–86], photosynthetic membranes [69, 81], or different adaptive feeding strategies [65] to name a few. In a presently changing climate, the rapid elevation of atmospheric CO_2_ and temperature will have severe consequences for the physiological performance of dinoflagellates. Given the importance of symbiont abundance in a symbiotic relationship [24, 87, 88], understanding the factors that contribute to different growth patterns, both inside and outside of symbiotic associations, is essential.

## Supporting information

S1 Table Gelman-Rubin potential scale reduction factor (PSRF) and upper CI for all parameters denoted by treatment index. (CSV)

S2 Table Summary of parameter posterior sample differences between pairs of cultures. Rows marked with “*” in the “Sig” column have less than 2.5%, or more than 97.5% of the distribution greater than zero.
(CSV)

S3 Table Summary of parameter posterior sample differences between pairs of treatments. Rows marked with “*” in the “Sig” column have less than 2.5%, or more than 97.5% of the distribution greater than zero.
(CSV)

S1 Fig Gelman-Rubin shrink factors as a function of MCMC iterations for each parameter and treatment. Culture, temperature, and light levels for treatment indices are defined in the initial table.
(PDF)

S2 Fig MCMC traces of the posterior samples for all parameters in each treatment. (PDF)
