# Supplementary material for: Comparative growth rates of cultured marine dinoflagellates in the genus Symbiodinium and the effects of temperature and light
Source: PLoS One. 2017 Nov 29;12(11):e0187707. doi: 10.1371/journal.pone.0187707 (PMC5706665; doi:10.1371/journal.pone.0187707)
Supplement: S1 Fig — Culture, temperature, and light levels for treatment indices are defined in the initial table. (PDF) [file pone.0187707.s004.pdf]

Treatment Index	Culture	Temperature	Light
1	A194 (04−503)	T.18	L.049
2	A194 (04−503)	T.26	L.049
3	A194 (04−503)	T.26	L.117
4	A194 (04−503)	T.26	L.231
5	A194 (KB8)	T.18	L.049
6	A194 (KB8)	T.26	L.049
7	A194 (KB8)	T.26	L.117
8	A194 (KB8)	T.26	L.231
9	B184	T.18	L.049
10	B184	T.26	L.049
11	B184	T.26	L.117
12	B184	T.26	L.231
13	B224	T.18	L.049
14	B224	T.26	L.049
15	B224	T.26	L.117
16	B224	T.26	L.231
17	D206	T.18	L.049
18	D206	T.26	L.049
19	D206	T.26	L.117
20	D206	T.26	L.231

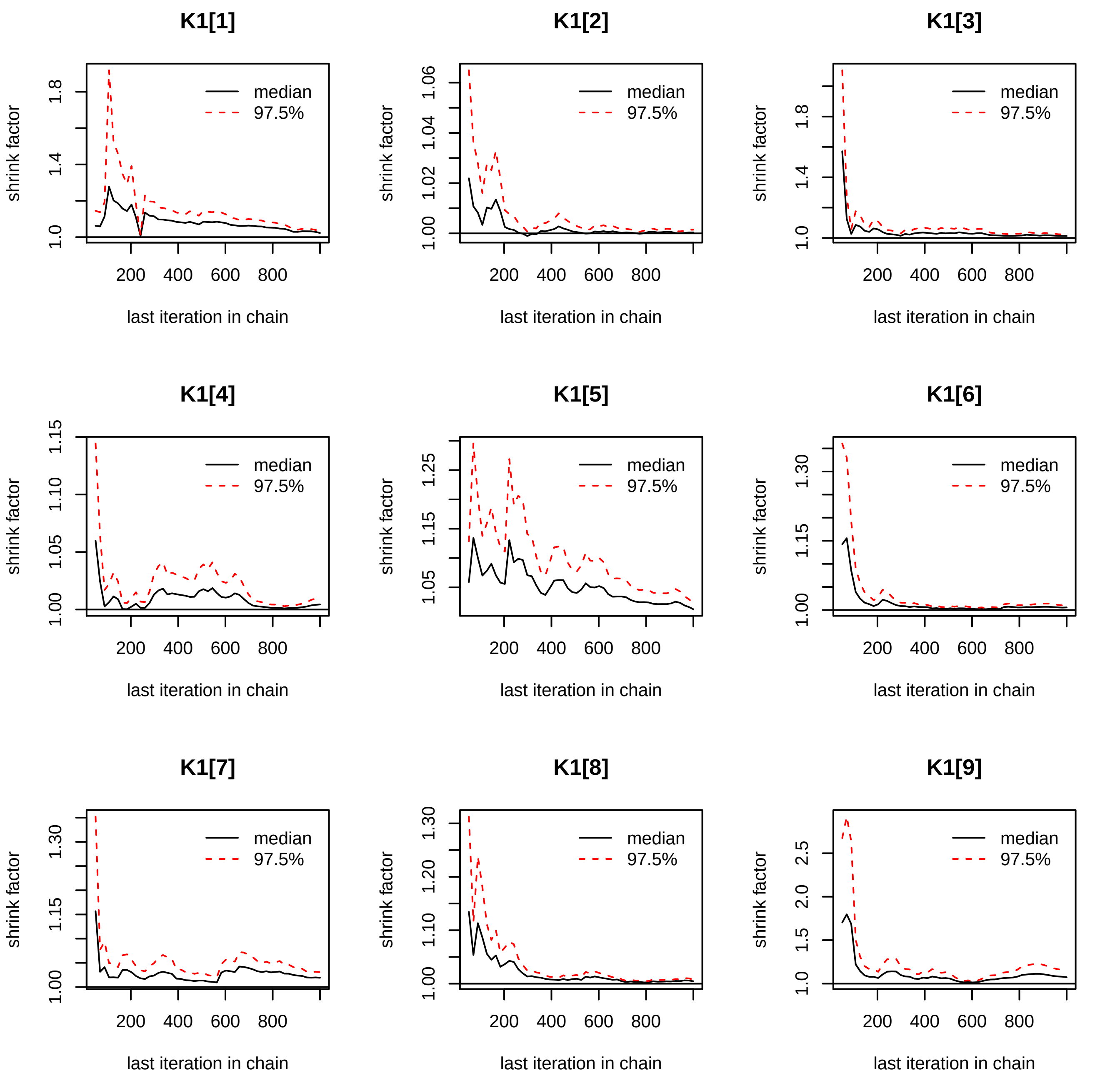
K1[1]
shrink factor
1.0
1.4
1.8
median
97.5%
200
400
600
800
last iteration in chain
K1[2]
shrink factor
1.00
1.02
1.04
1.06
median
97.5%
200
400
600
800
last iteration in chain
K1[3]
shrink factor
1.0
1.4
1.8
median
97.5%
200
400
600
800
last iteration in chain
K1[4]
shrink factor
1.00
1.05
1.10
1.15
median
97.5%
200
400
600
800
last iteration in chain
K1[5]
shrink factor
1.05
1.15
1.25
median
97.5%
200
400
600
800
last iteration in chain
K1[6]
shrink factor
1.00
1.15
1.30
median
97.5%
200
400
600
800
last iteration in chain
K1[7]
shrink factor
1.00
1.15
1.30
median
97.5%
200
400
600
800
last iteration in chain
K1[8]
shrink factor
1.00
1.10
1.20
1.30
median
97.5%
200
400
600
800
last iteration in chain
K1[9]
shrink factor
1.0
1.5
2.0
2.5
median
97.5%
200
400
600
800
last iteration in chain

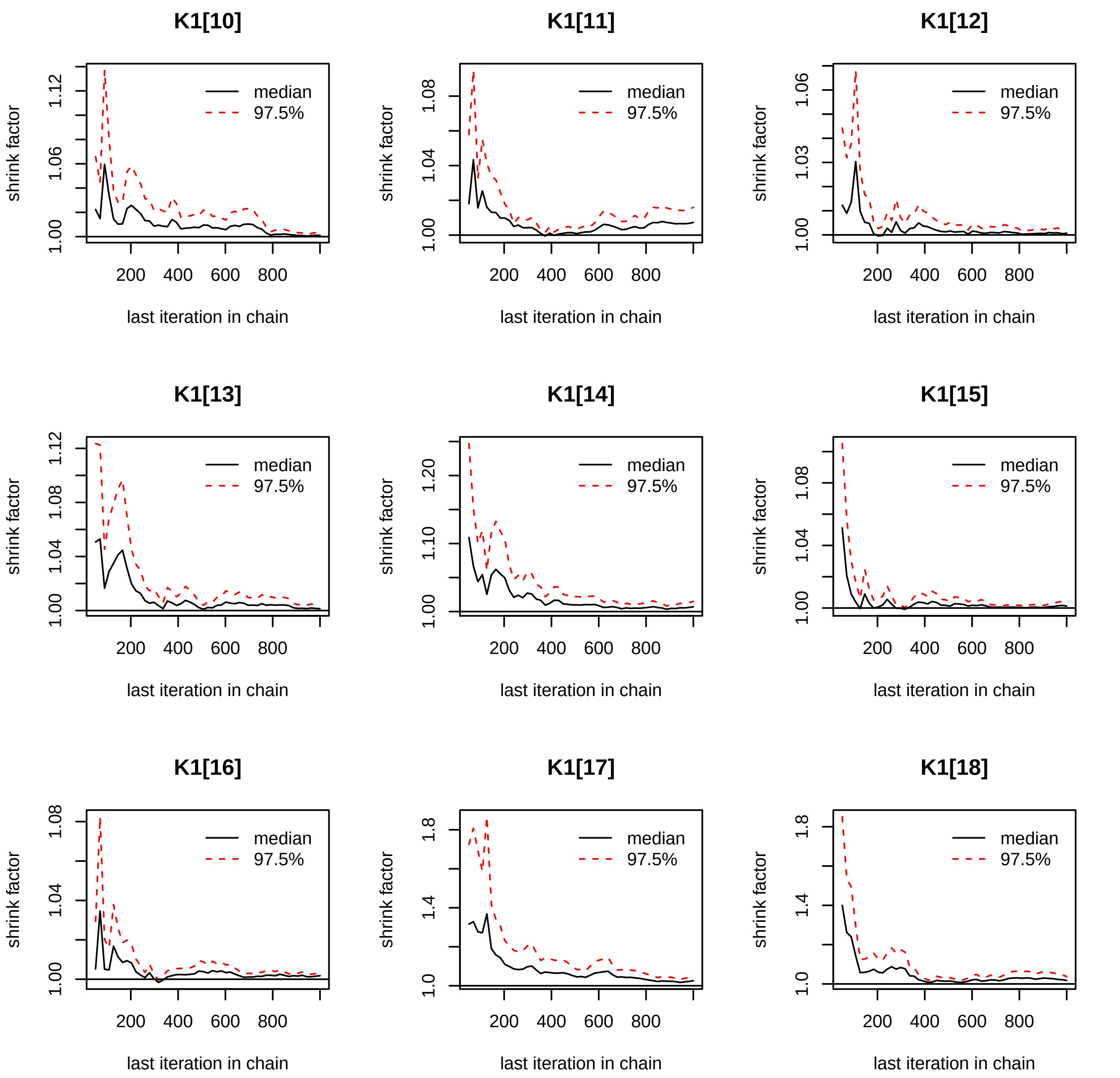
K1[10]
K1[11]
K1[12]
K1[13]
K1[14]
K1[15]
K1[16]
K1[17]
K1[18]
median
97.5%
shrink factor
last iteration in chain
200
400
600
800
1.00
1.06
1.12
1.04
1.08
1.03
1.10
1.20
1.0
1.4
1.8

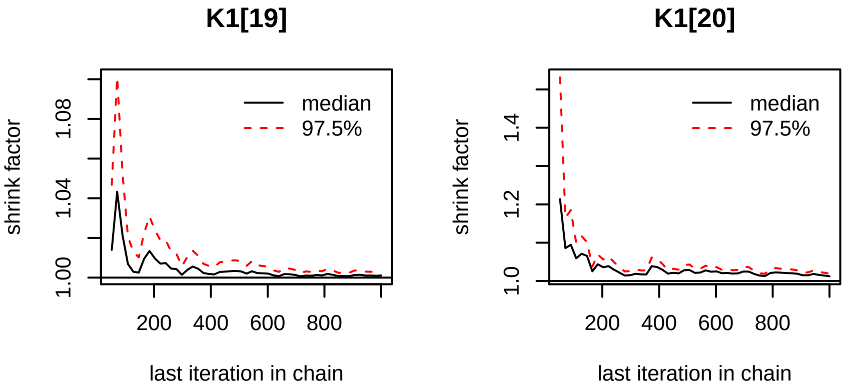
K1[19]
K1[20]
median
97.5%
shrink factor
1.00
1.04
1.08
1.0
1.2
1.4
200
400
600
800
last iteration in chain

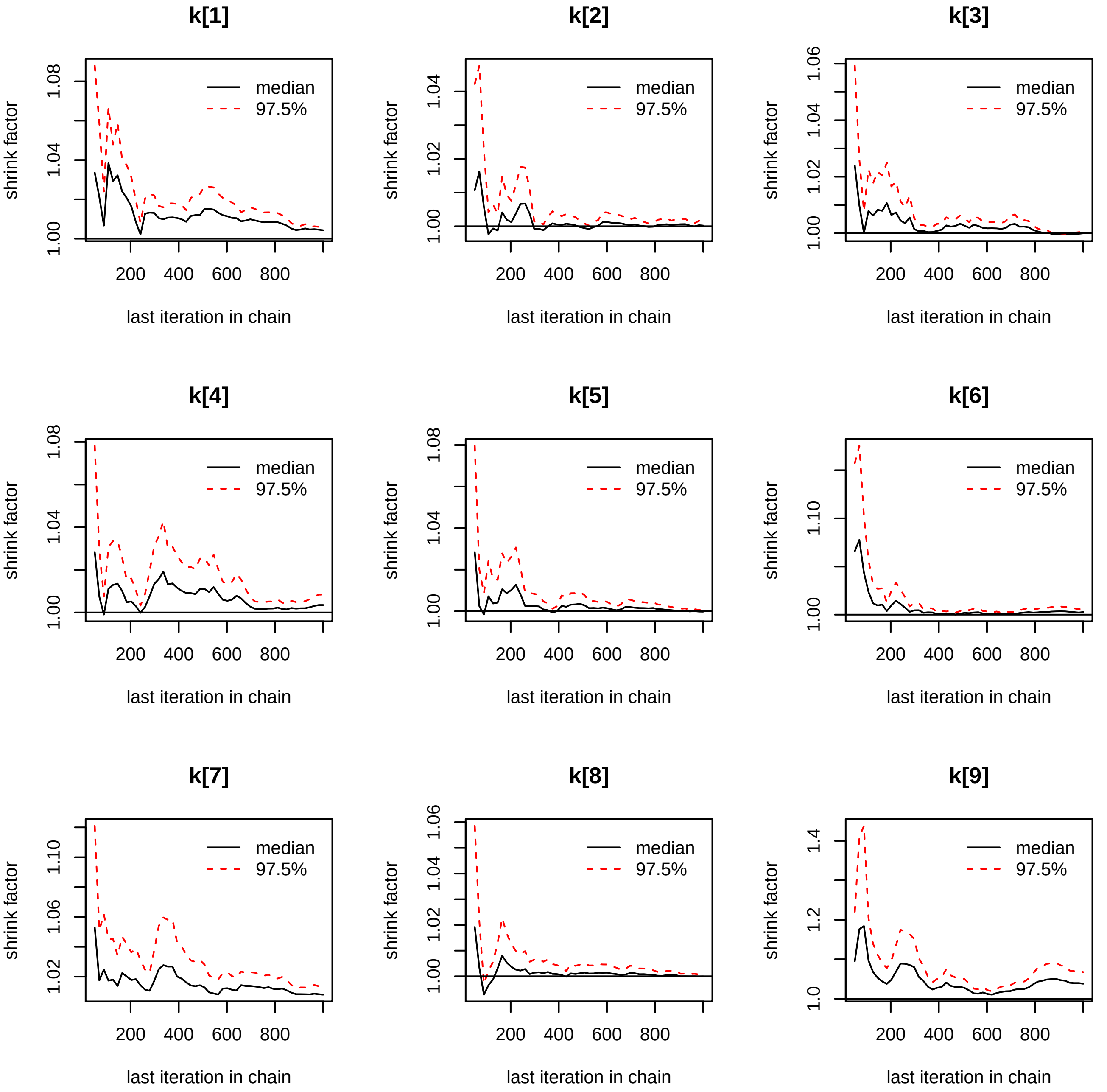
k[1]
k[2]
k[3]
k[4]
k[5]
k[6]
k[7]
k[8]
k[9]
median
97.5%
shrink factor
last iteration in chain
200
400
600
800

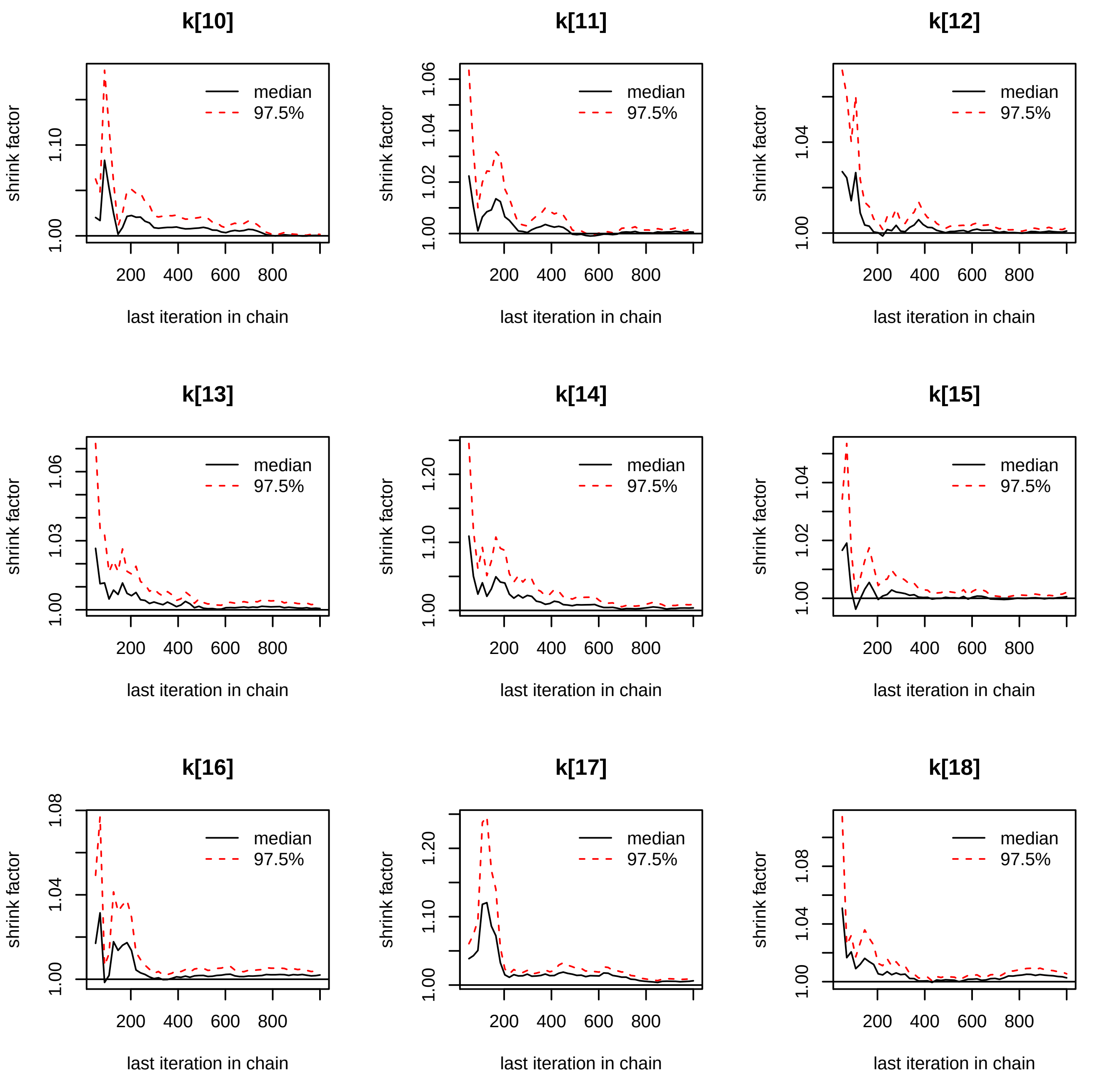
k[10]
k[11]
k[12]
k[13]
k[14]
k[15]
k[16]
k[17]
k[18]
median
97.5%
shrink factor
last iteration in chain
200
400
600
800
1.00
1.10
1.02
1.04
1.06
1.03
1.20
1.08

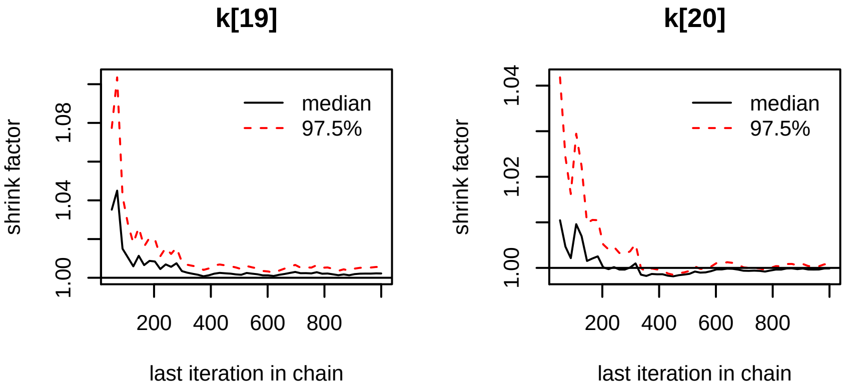

k[19]
median
97.5%
shrink factor
1.00
1.04
1.08
200
400
600
800
last iteration in chain
k[20]
median
97.5%
shrink factor
1.00
1.02
1.04
200
400
600
800
last iteration in chain

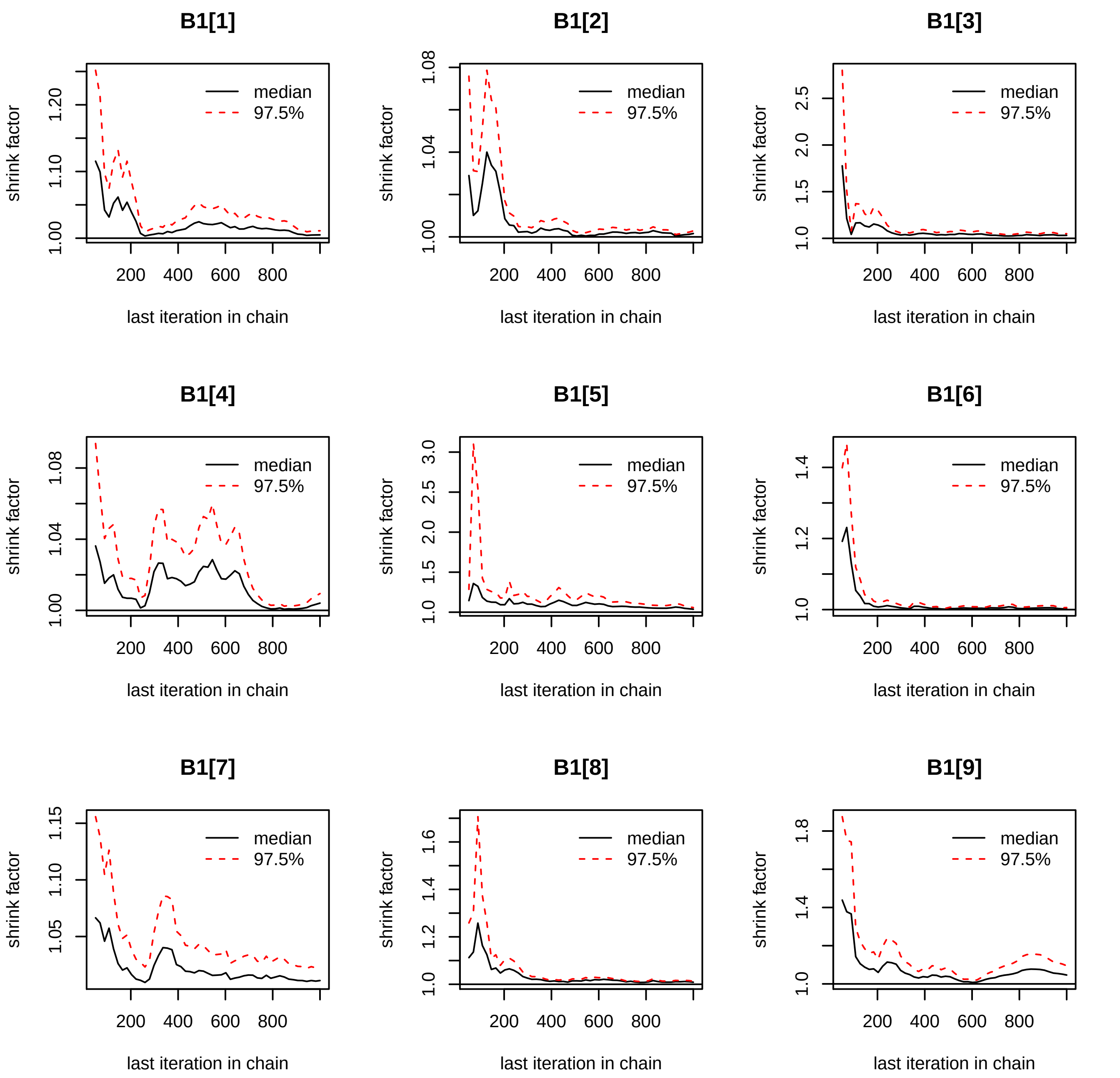

B1[1]
B1[2]
B1[3]
B1[4]
B1[5]
B1[6]
B1[7]
B1[8]
B1[9]
median
97.5%
shrink factor
last iteration in chain
200
400
600
800
1.00
1.10
1.20
1.04
1.08
1.0
1.5
2.0
2.5
3.0
1.2
1.4
1.6
1.05
1.15
1.8

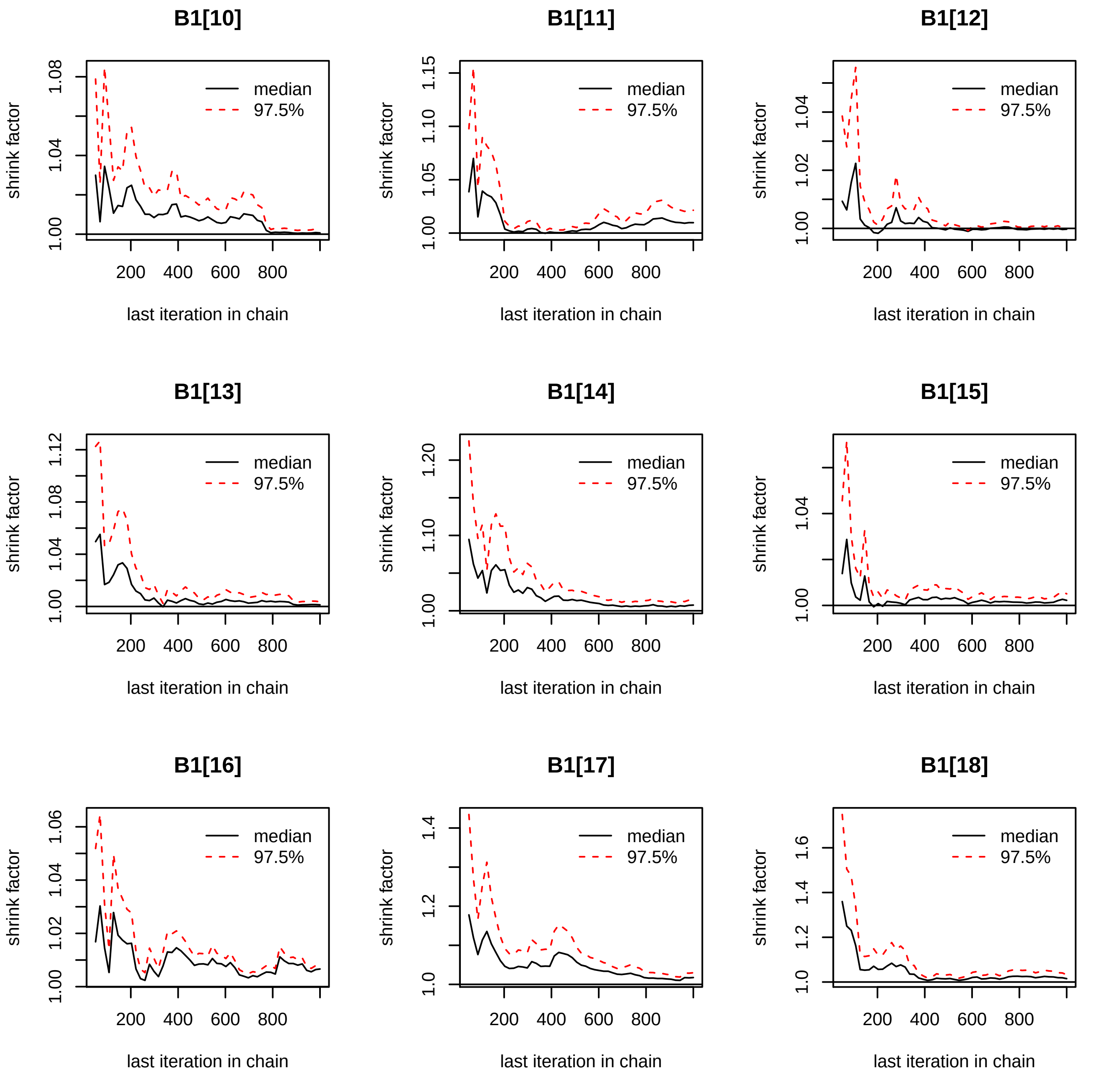
B1[10]
shrink factor
1.00
1.04
1.08
median
97.5%
200
400
600
800
last iteration in chain
B1[11]
shrink factor
1.00
1.05
1.10
1.15
median
97.5%
200
400
600
800
last iteration in chain
B1[12]
shrink factor
1.00
1.02
1.04
median
97.5%
200
400
600
800
last iteration in chain
B1[13]
shrink factor
1.00
1.04
1.08
1.12
median
97.5%
200
400
600
800
last iteration in chain
B1[14]
shrink factor
1.00
1.10
1.20
median
97.5%
200
400
600
800
last iteration in chain
B1[15]
shrink factor
1.00
1.04
median
97.5%
200
400
600
800
last iteration in chain
B1[16]
shrink factor
1.00
1.02
1.04
1.06
median
97.5%
200
400
600
800
last iteration in chain
B1[17]
shrink factor
1.0
1.2
1.4
median
97.5%
200
400
600
800
last iteration in chain
B1[18]
shrink factor
1.0
1.2
1.4
1.6
median
97.5%
200
400
600
800
last iteration in chain

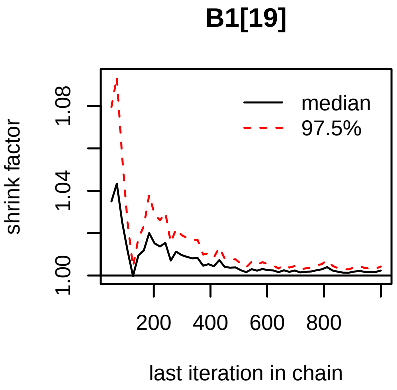

B1[19]
median
97.5%
shrink factor
1.00
1.04
1.08
200
400
600
800
last iteration in chain

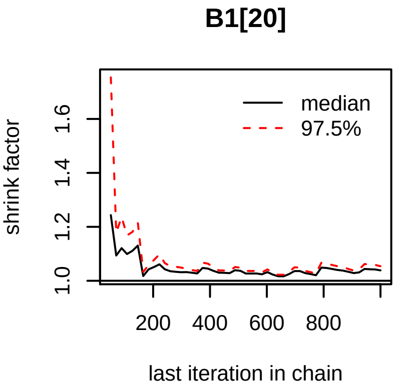

B1[20]
median
97.5%
shrink factor
1.0
1.2
1.4
1.6
200
400
600
800
last iteration in chain

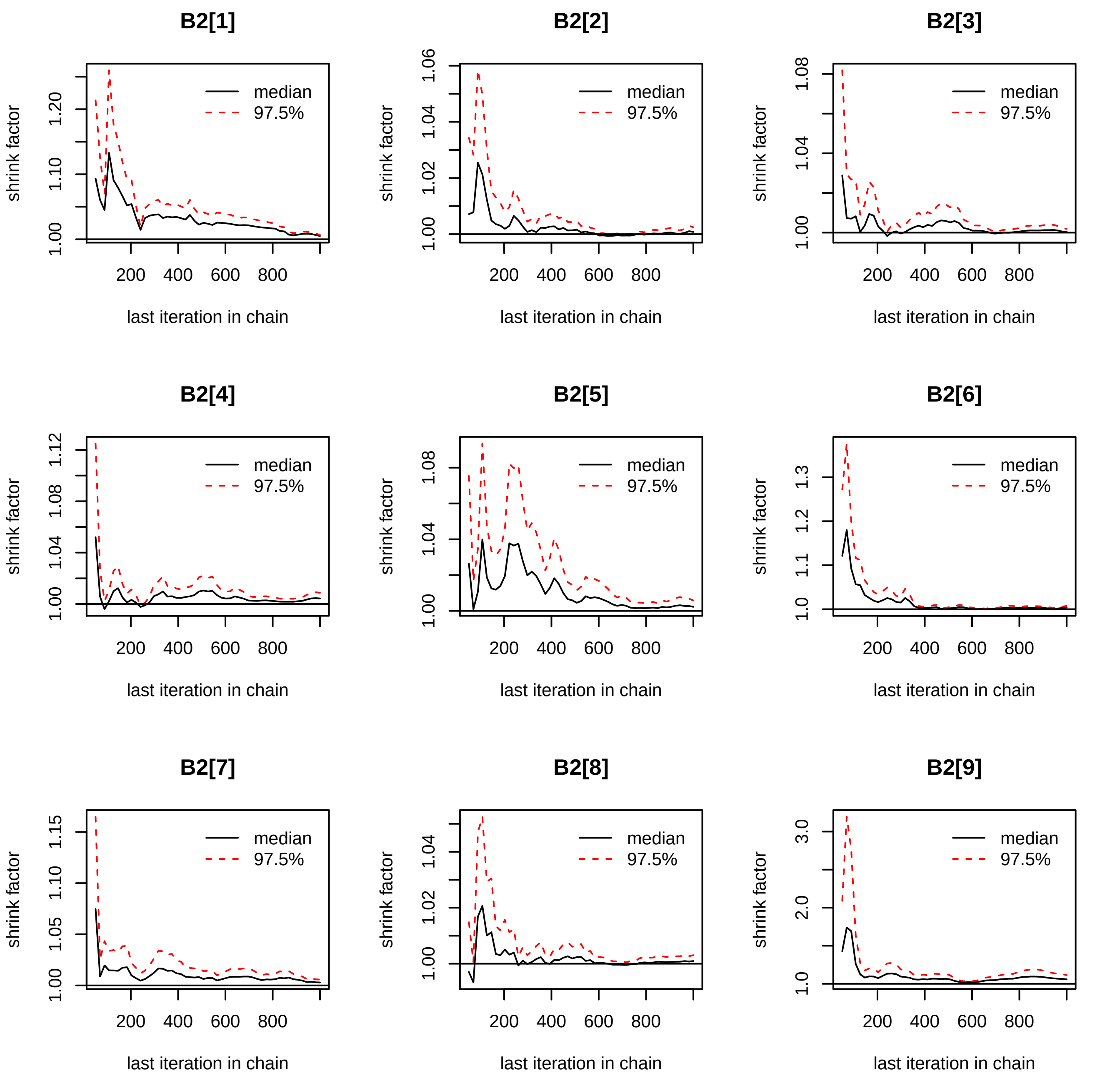
B2[1]
B2[2]
B2[3]
B2[4]
B2[5]
B2[6]
B2[7]
B2[8]
B2[9]
shrink factor
last iteration in chain
median
97.5%
200
400
600
800
1.00
1.10
1.20
1.02
1.04
1.06
1.08
1.12
1.0
1.1
1.2
1.3
1.05
1.15
2.0
3.0

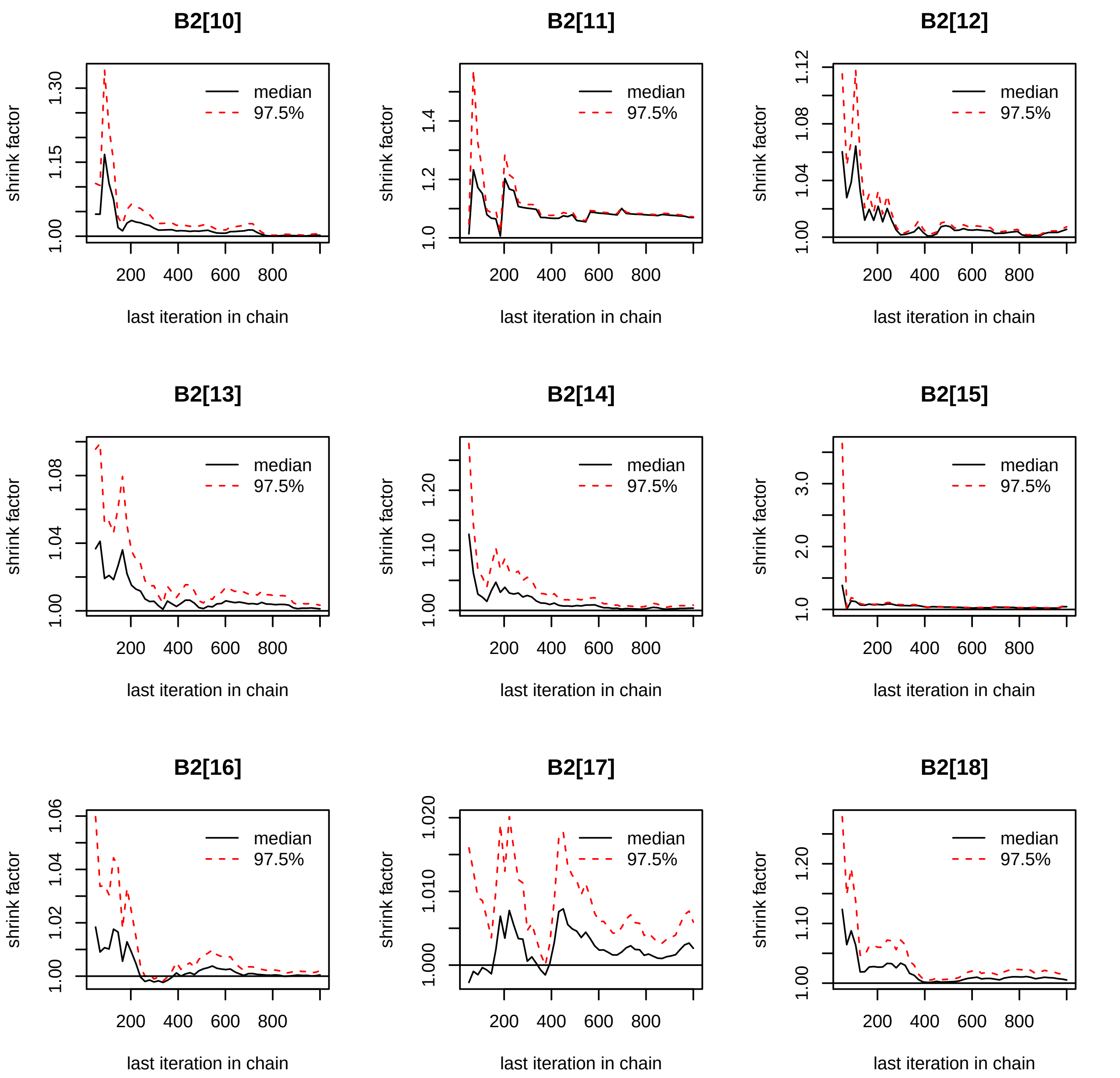
B2[10]
B2[11]
B2[12]
B2[13]
B2[14]
B2[15]
B2[16]
B2[17]
B2[18]
median
97.5%
shrink factor
last iteration in chain
200
400
600
800
1.00
1.15
1.30
1.0
1.2
1.4
1.00
1.04
1.08
1.12
1.00
1.04
1.08
1.00
1.10
1.20
1.0
2.0
3.0
1.00
1.02
1.04
1.06
1.000
1.010
1.020
1.00
1.10
1.20

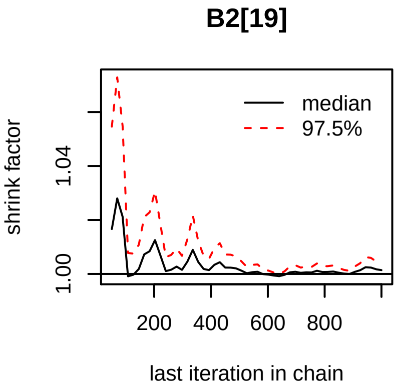
B2[19]
shrink factor
1.00
1.04
200
400
600
800
median
97.5%
last iteration in chain

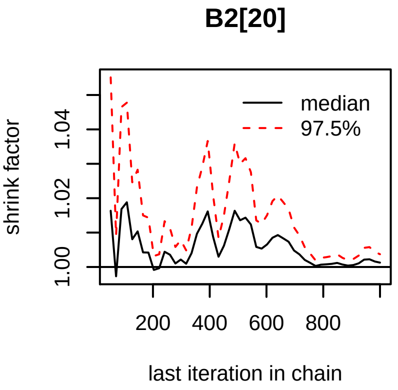
B2[20]
shrink factor
1.00
1.02
1.04
200
400
600
800
median
97.5%
last iteration in chain

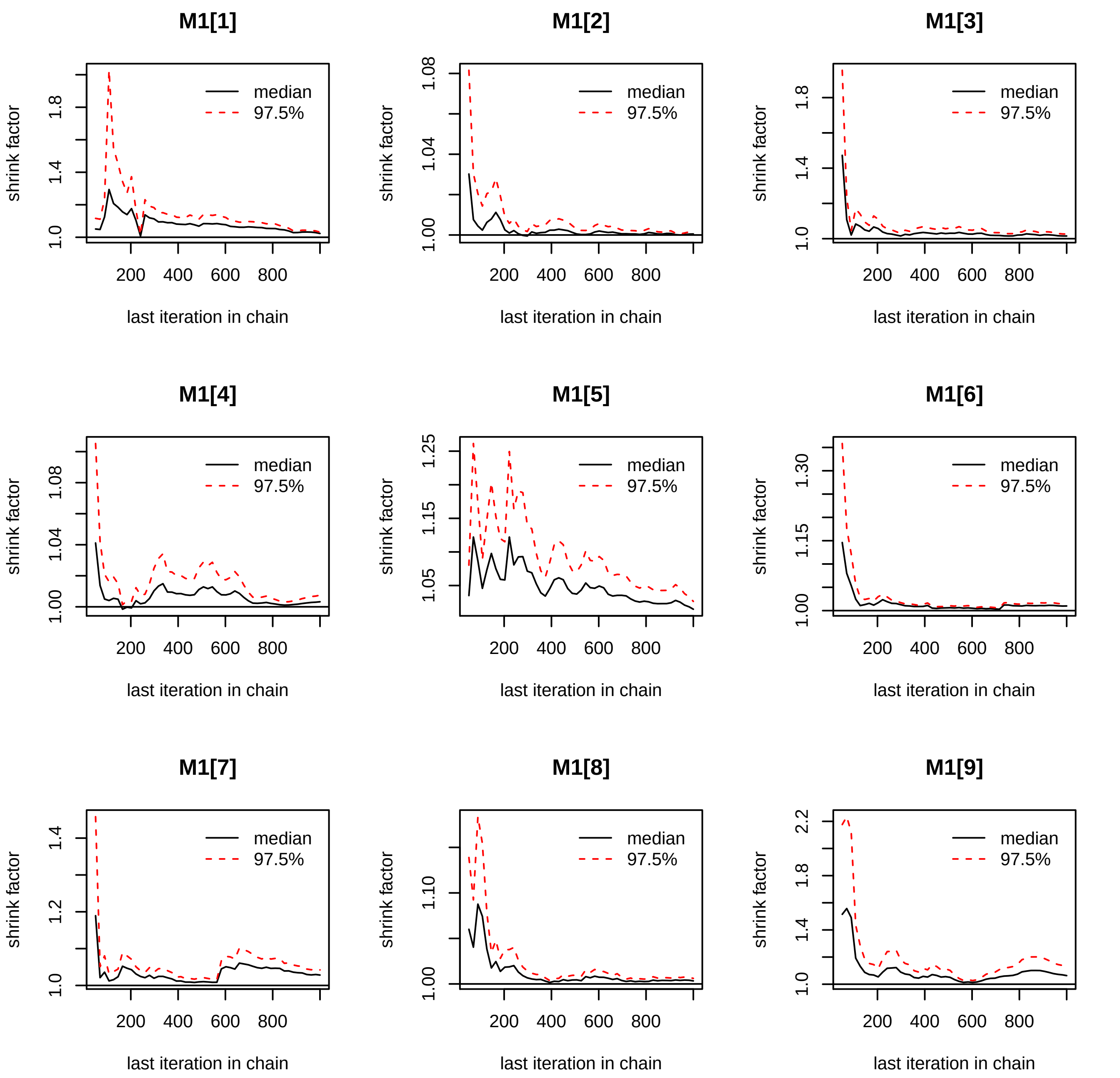

M1[1]
M1[2]
M1[3]
M1[4]
M1[5]
M1[6]
M1[7]
M1[8]
M1[9]
median
97.5%
shrink factor
last iteration in chain
200
400
600
800

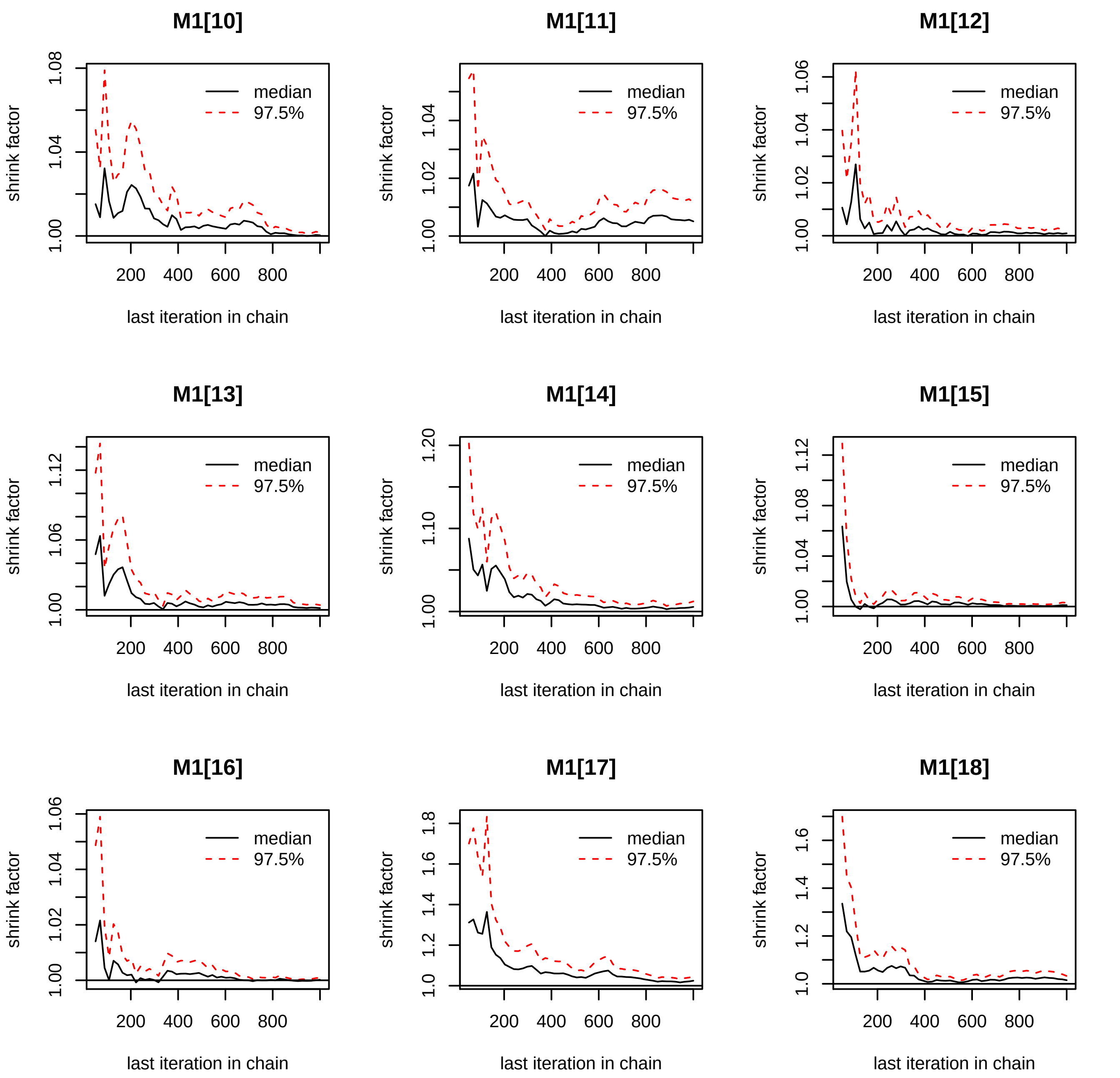
M1[10]
M1[11]
M1[12]
M1[13]
M1[14]
M1[15]
M1[16]
M1[17]
M1[18]
shrink factor
last iteration in chain
median
97.5%
200
400
600
800
1.00
1.02
1.04
1.06
1.08
1.10
1.12
1.20
1.0
1.2
1.4
1.6
1.8

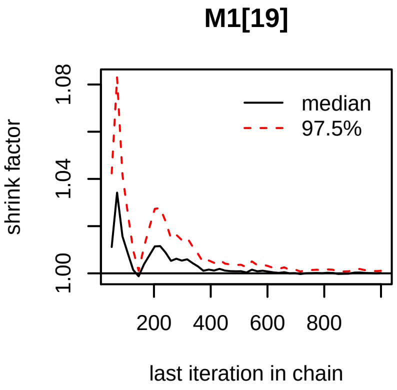
M1[19]
median
97.5%
shrink factor
1.00
1.04
1.08
200
400
600
800
last iteration in chain

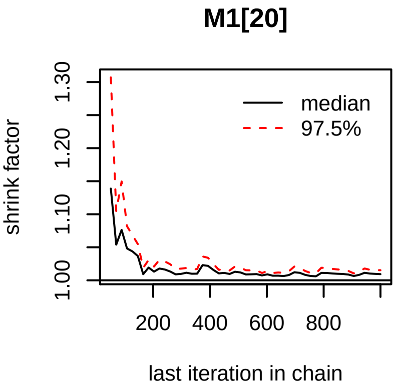
M1[20]
median
97.5%
shrink factor
1.00
1.10
1.20
1.30
200
400
600
800
last iteration in chain

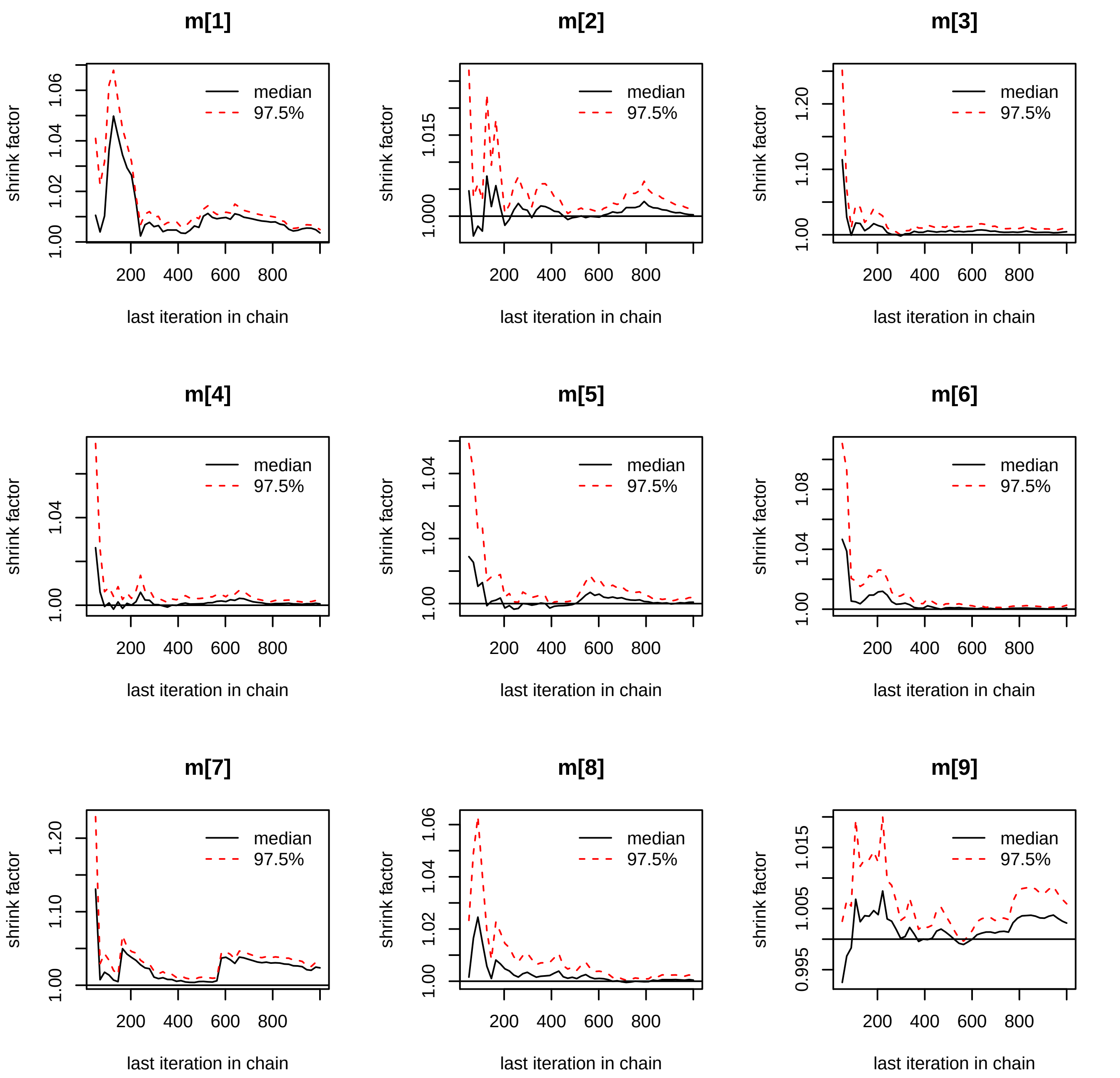
m[1]
m[2]
m[3]
m[4]
m[5]
m[6]
m[7]
m[8]
m[9]
median
97.5%
shrink factor
last iteration in chain
200
400
600
800
1.00
1.02
1.04
1.06
1.000
1.015
1.10
1.20
1.08
0.995
1.005

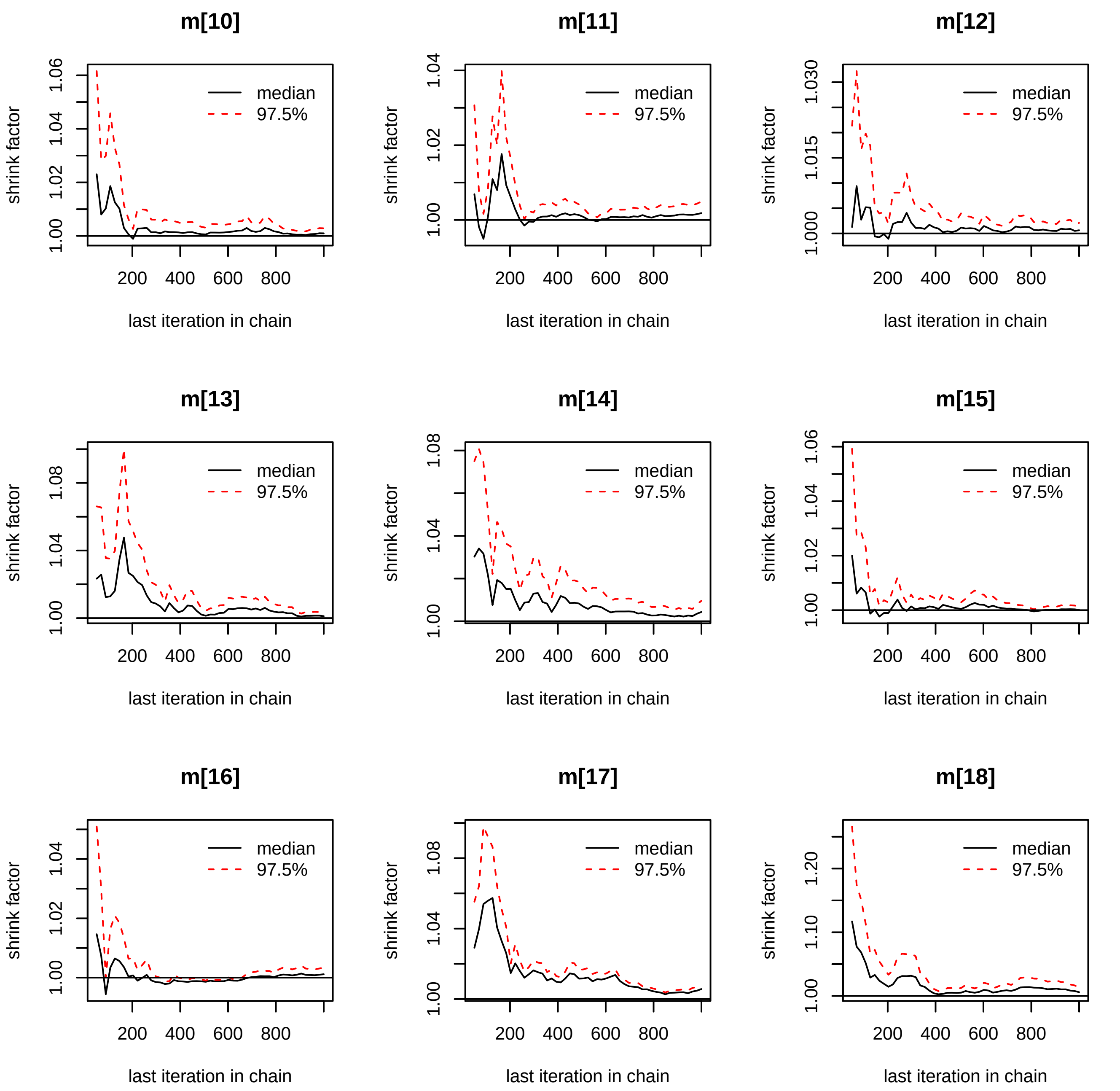

m[10]
m[11]
m[12]
m[13]
m[14]
m[15]
m[16]
m[17]
m[18]
shrink factor
last iteration in chain
median
97.5%
200
400
600
800
1.00
1.02
1.04
1.06
1.08
1.000
1.015
1.030
1.10
1.20

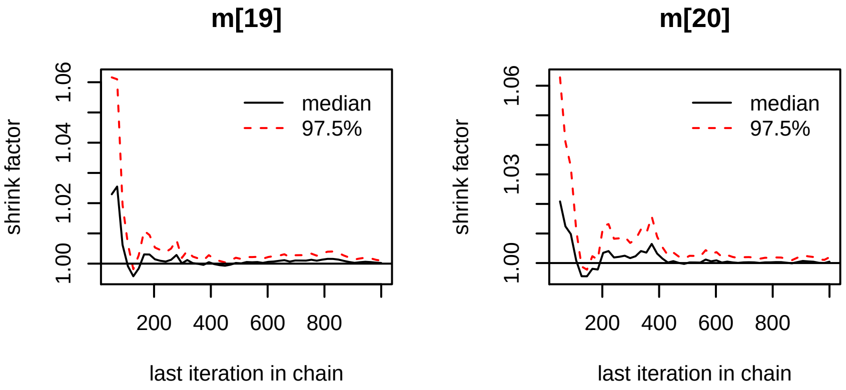
m[19]
m[20]
median
97.5%
shrink factor
1.00
1.02
1.04
1.06
1.03
200
400
600
800
last iteration in chain
