# Supplementary material for: Comparative growth rates of cultured marine dinoflagellates in the genus Symbiodinium and the effects of temperature and light
Source: PLoS One. 2017 Nov 29;12(11):e0187707. doi: 10.1371/journal.pone.0187707 (PMC5706665; doi:10.1371/journal.pone.0187707)
Supplement: S2 Fig — (PDF) [file pone.0187707.s005.pdf]

a) A194 (04−503) / T.18 / L.049

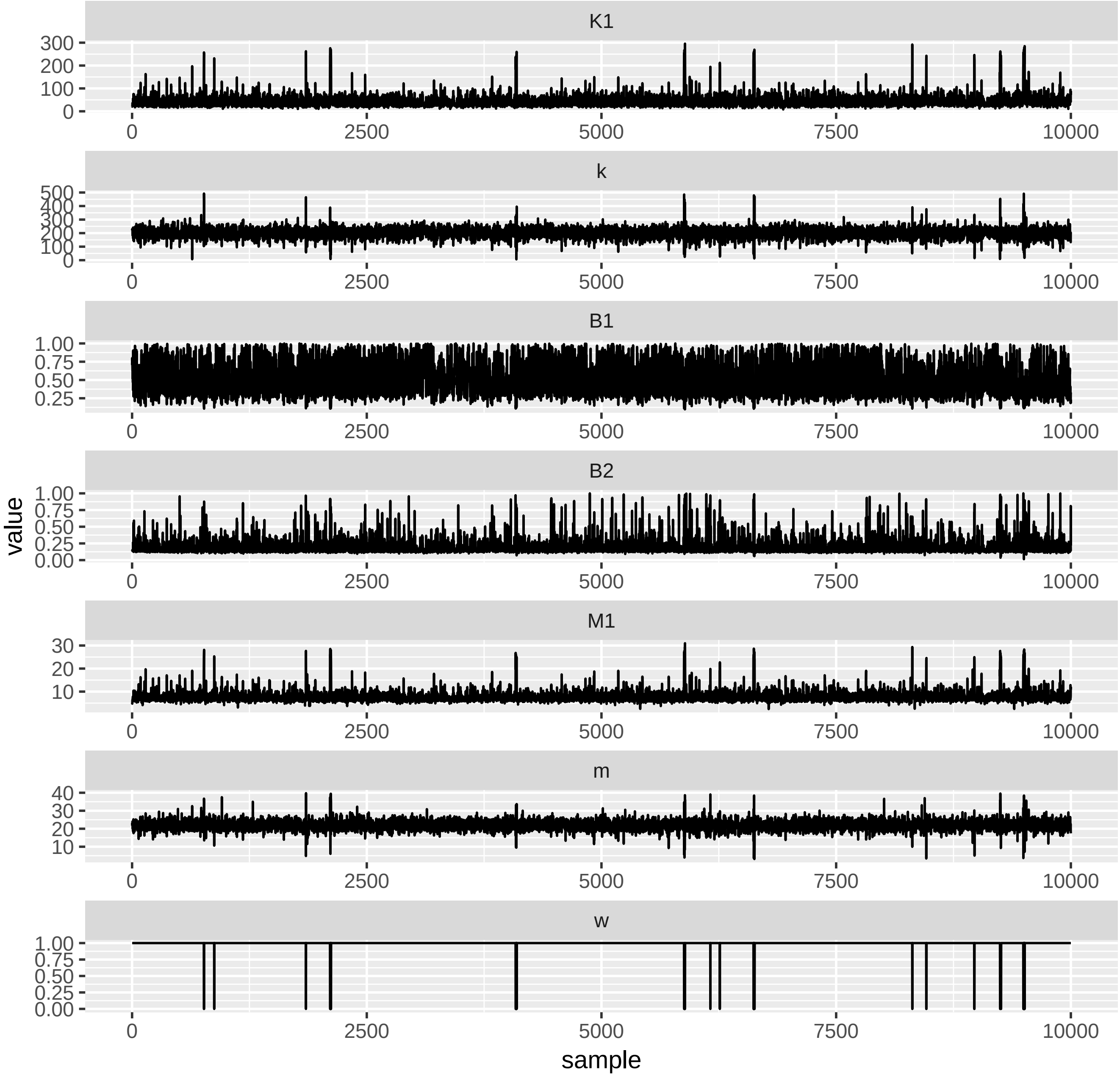

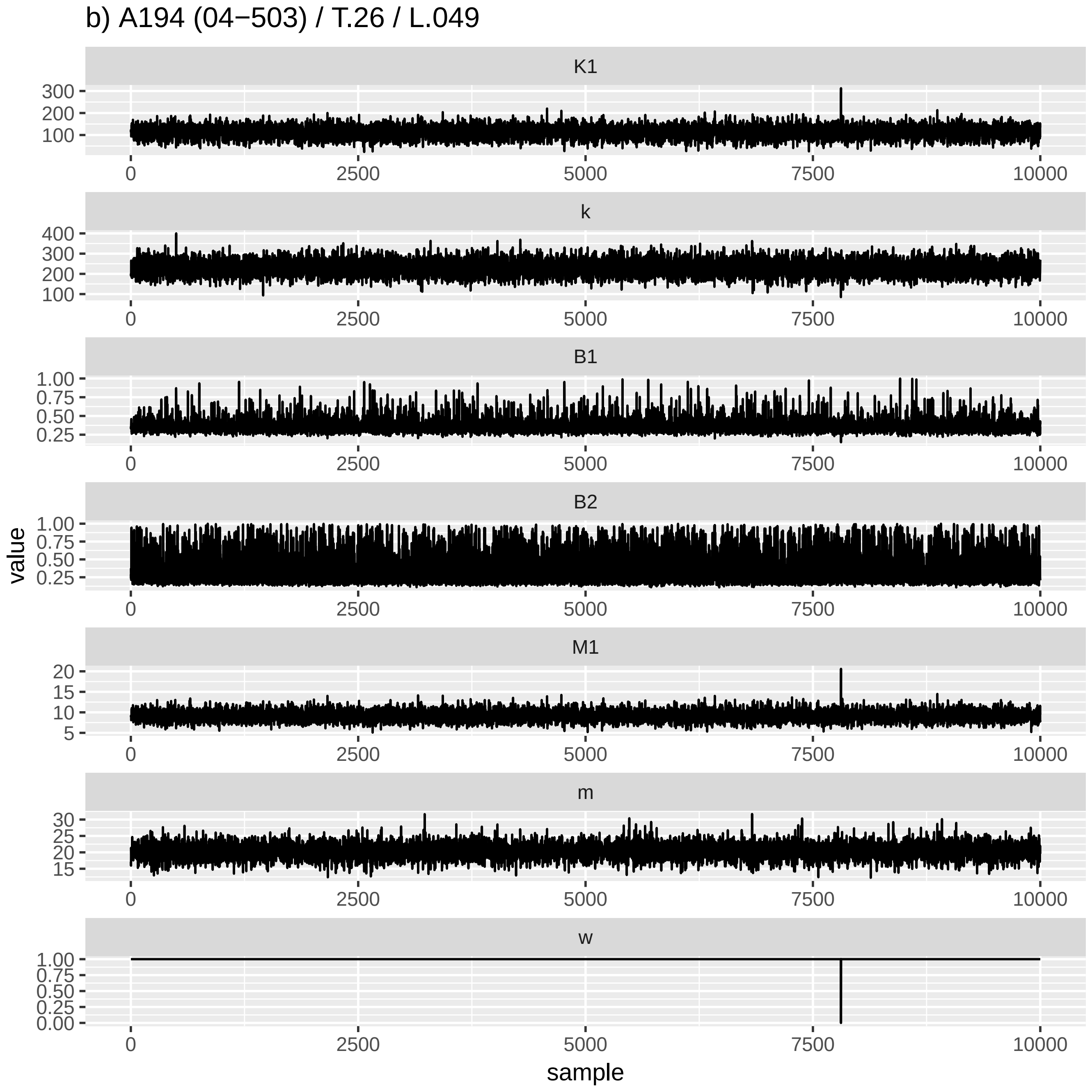
b) A194 (04−503) / T.26 / L.049
K1
k
B1
B2
M1
m
w
value
sample
0
2500
5000
7500
10000

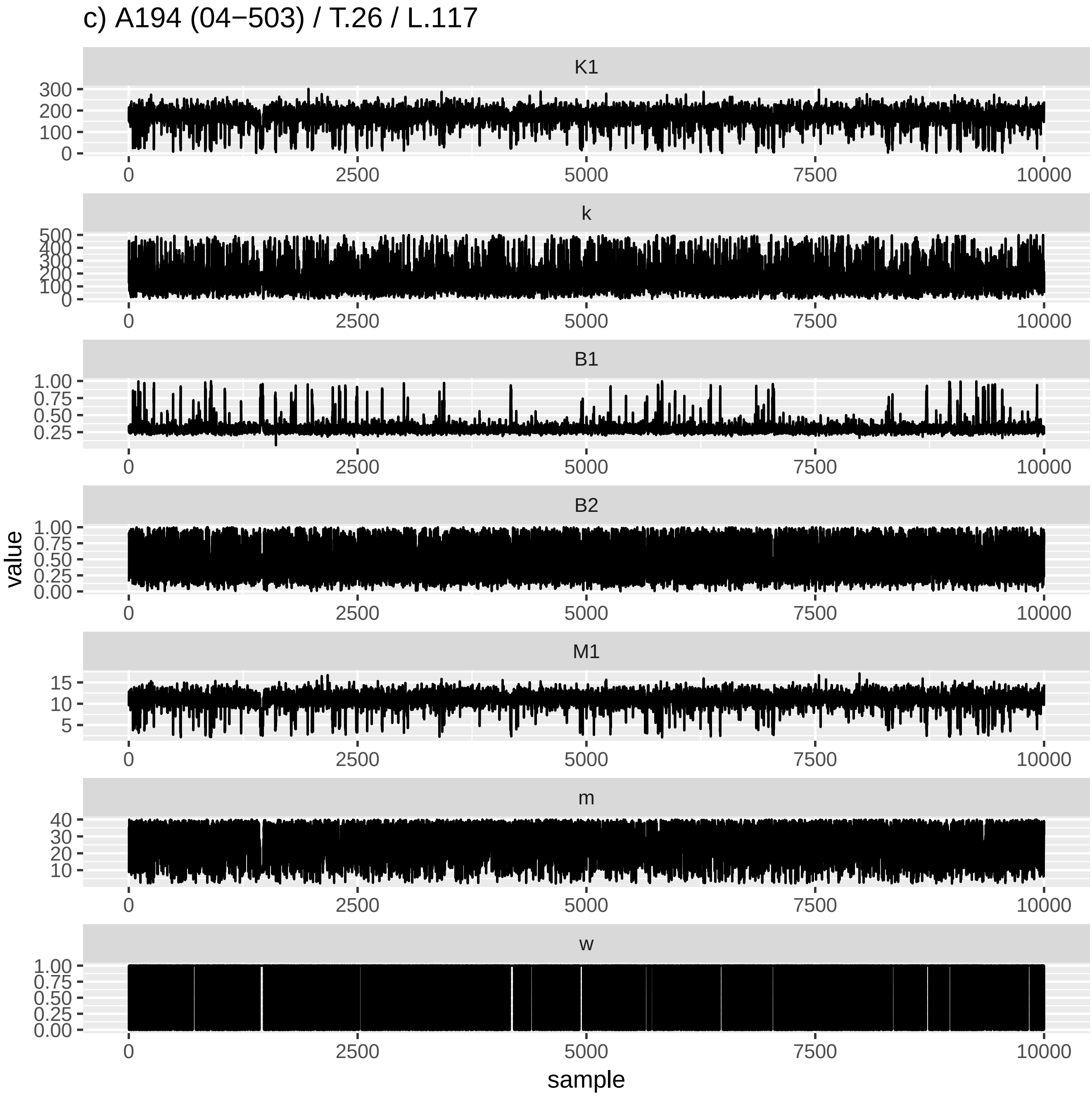
c) A194 (04−503) / T.26 / L.117
K1
k
B1
B2
M1
m
w
value
sample
0
2500
5000
7500
10000
0
100
200
300
400
500
0.25
0.50
0.75
1.00
0.00
5
10
15
20
30
40

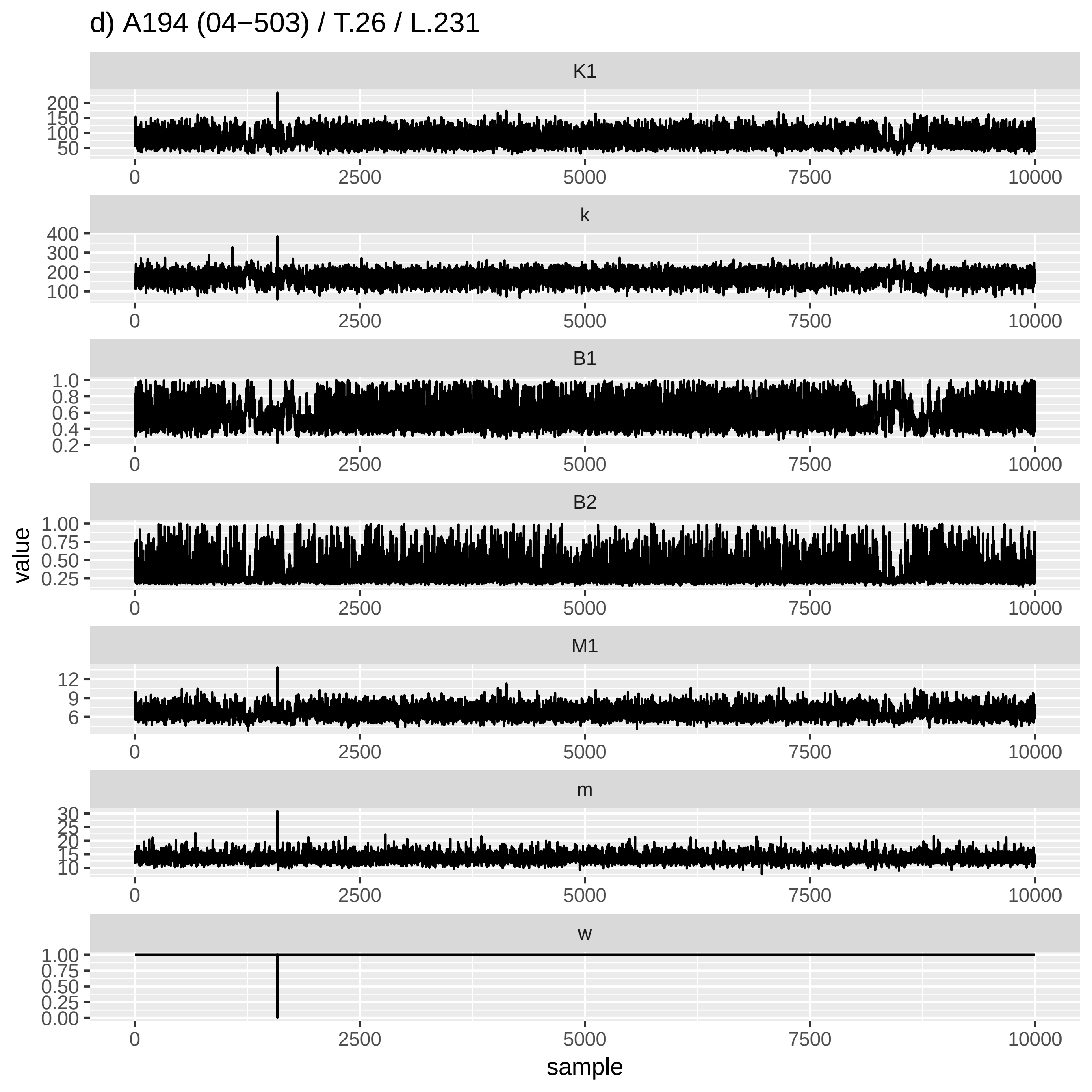

d) A194 (04−503) / T.26 / L.231
K1
k
B1
B2
M1
m
w
value
sample
0
2500
5000
7500
10000

e) A194 (KB8) / T.18 / L.049

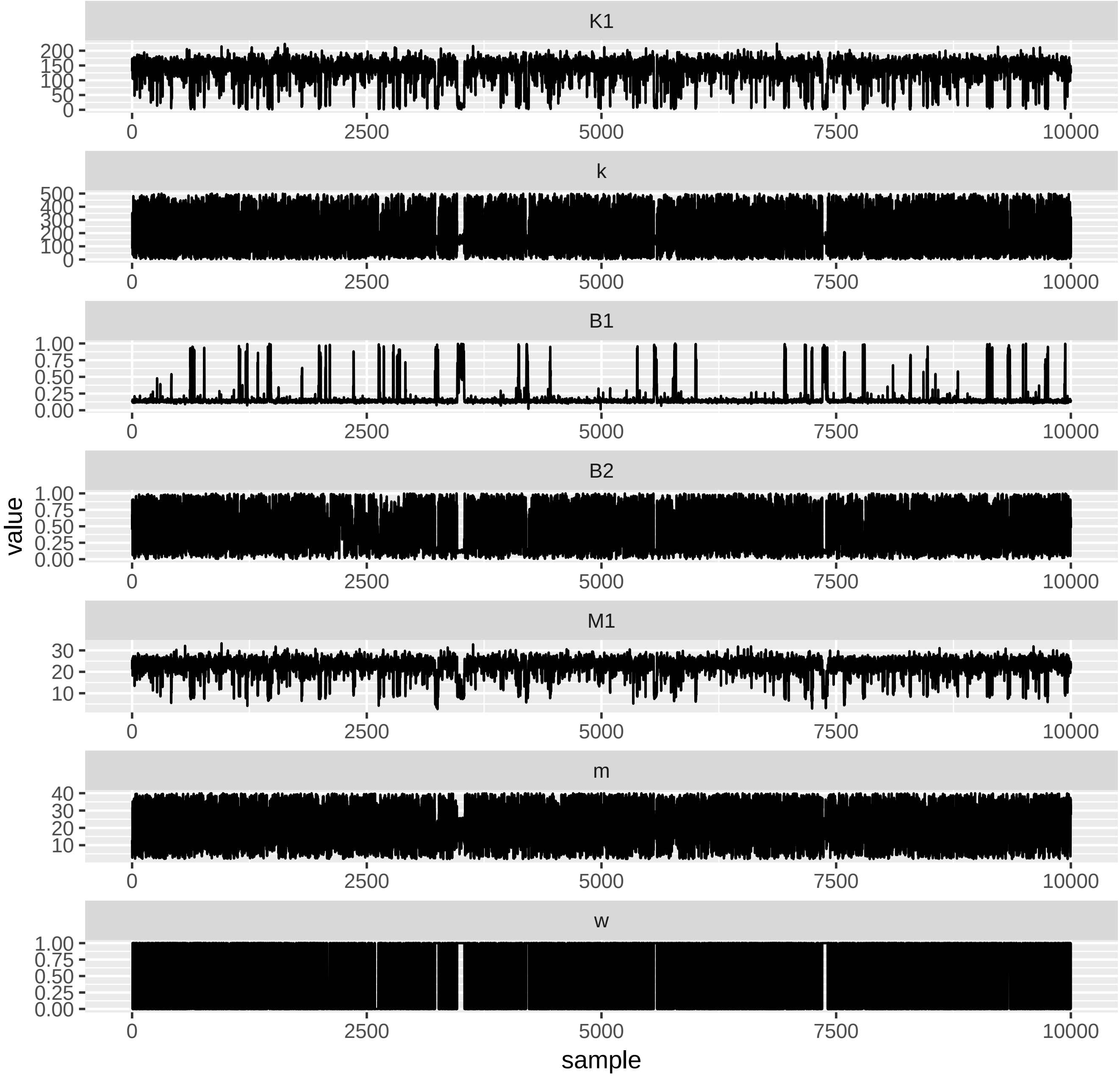

f) A194 (KB8) / T.26 / L.049

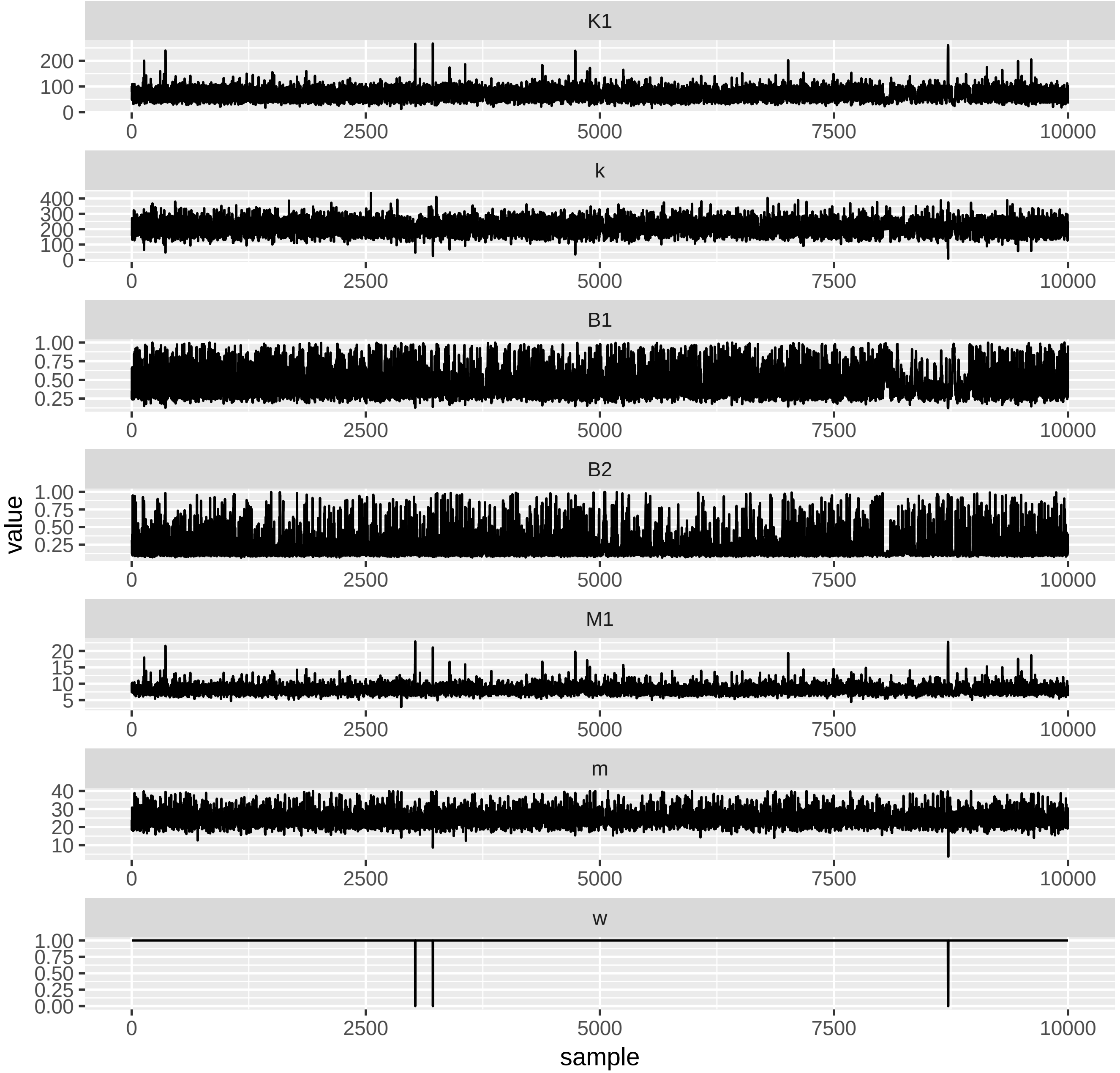

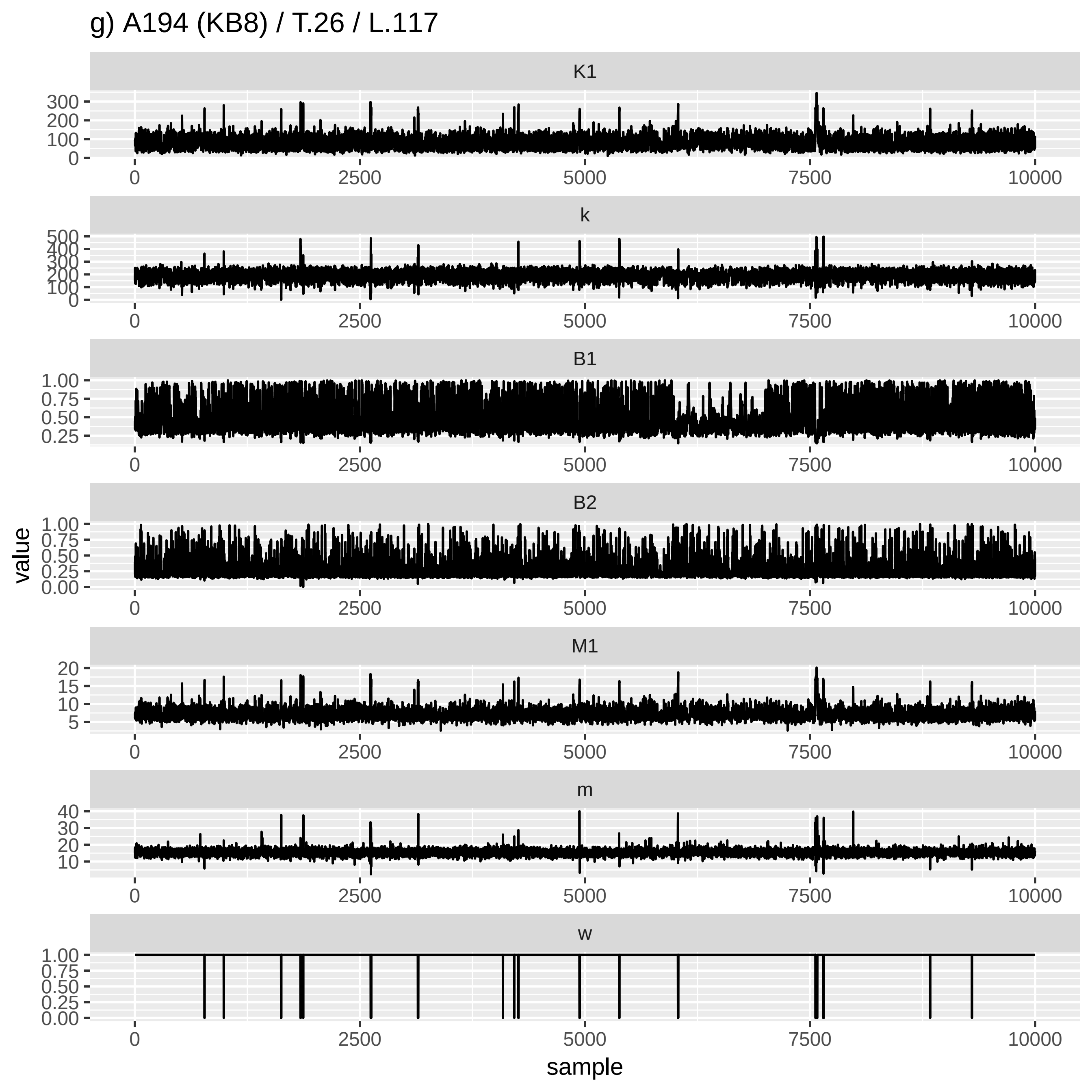
g) A194 (KB8) / T.26 / L.117
K1
k
B1
B2
M1
m
w
value
sample

h) A194 (KB8) / T.26 / L.231

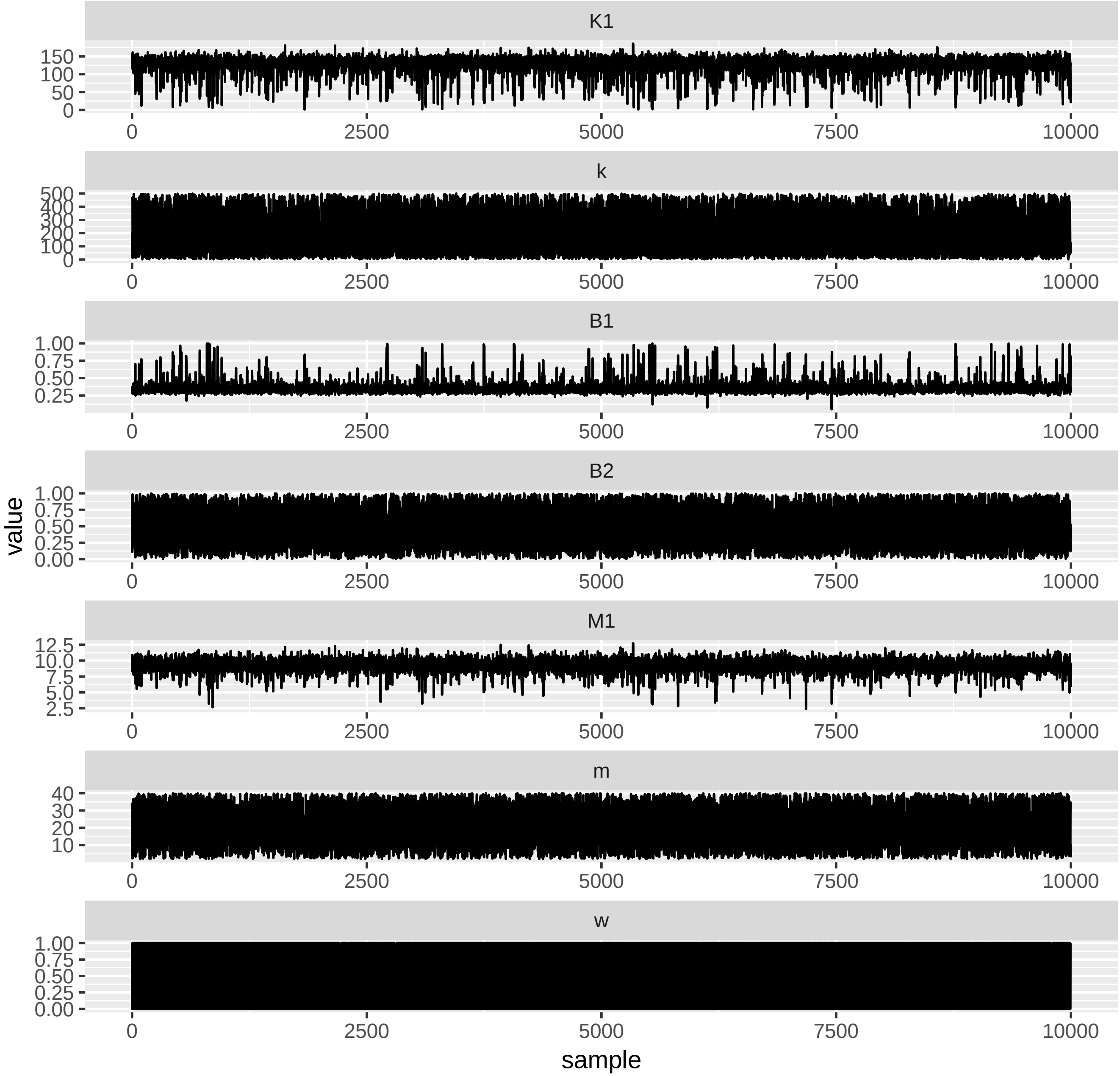

i) B184 / T.18 / L.049

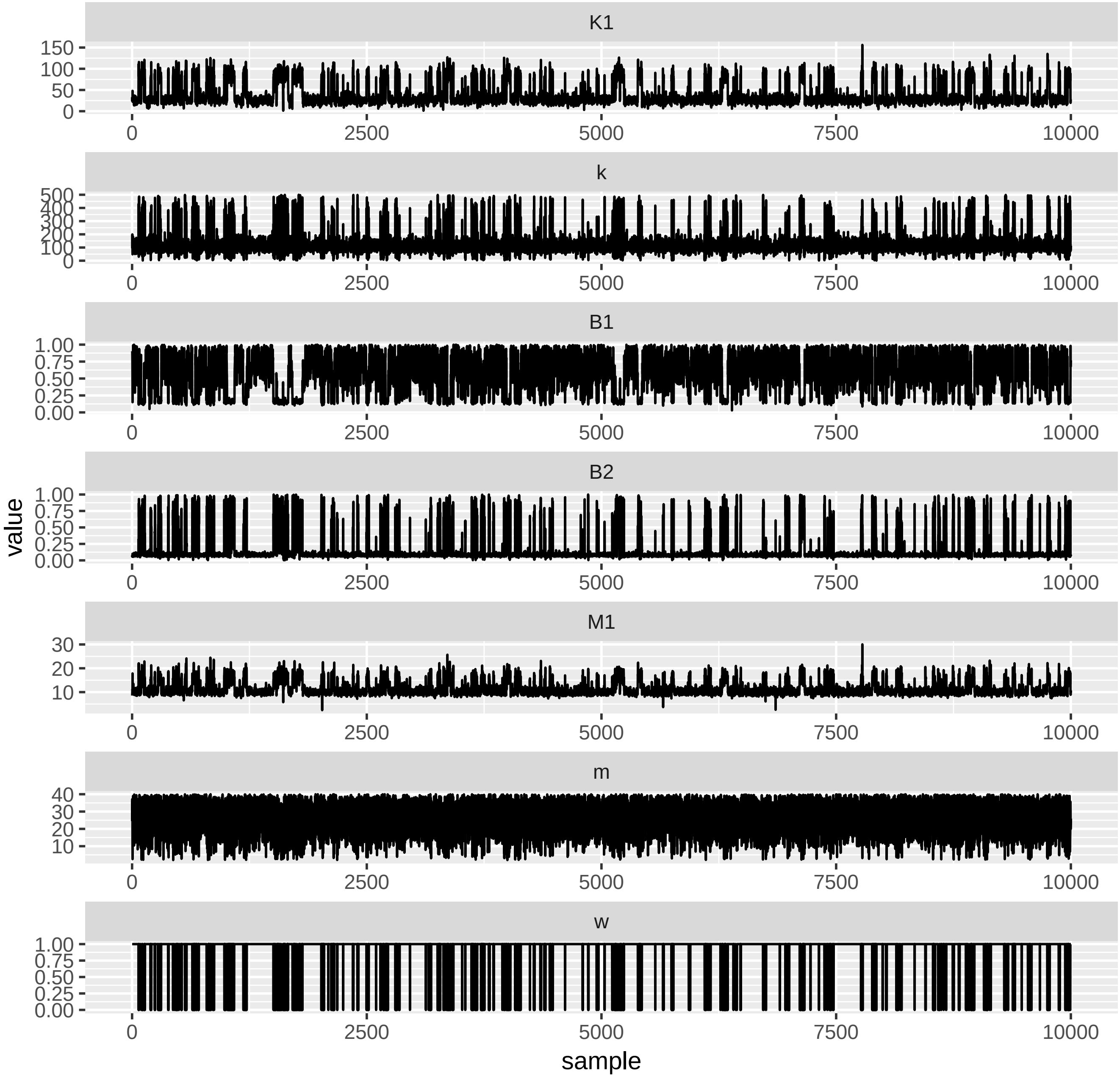

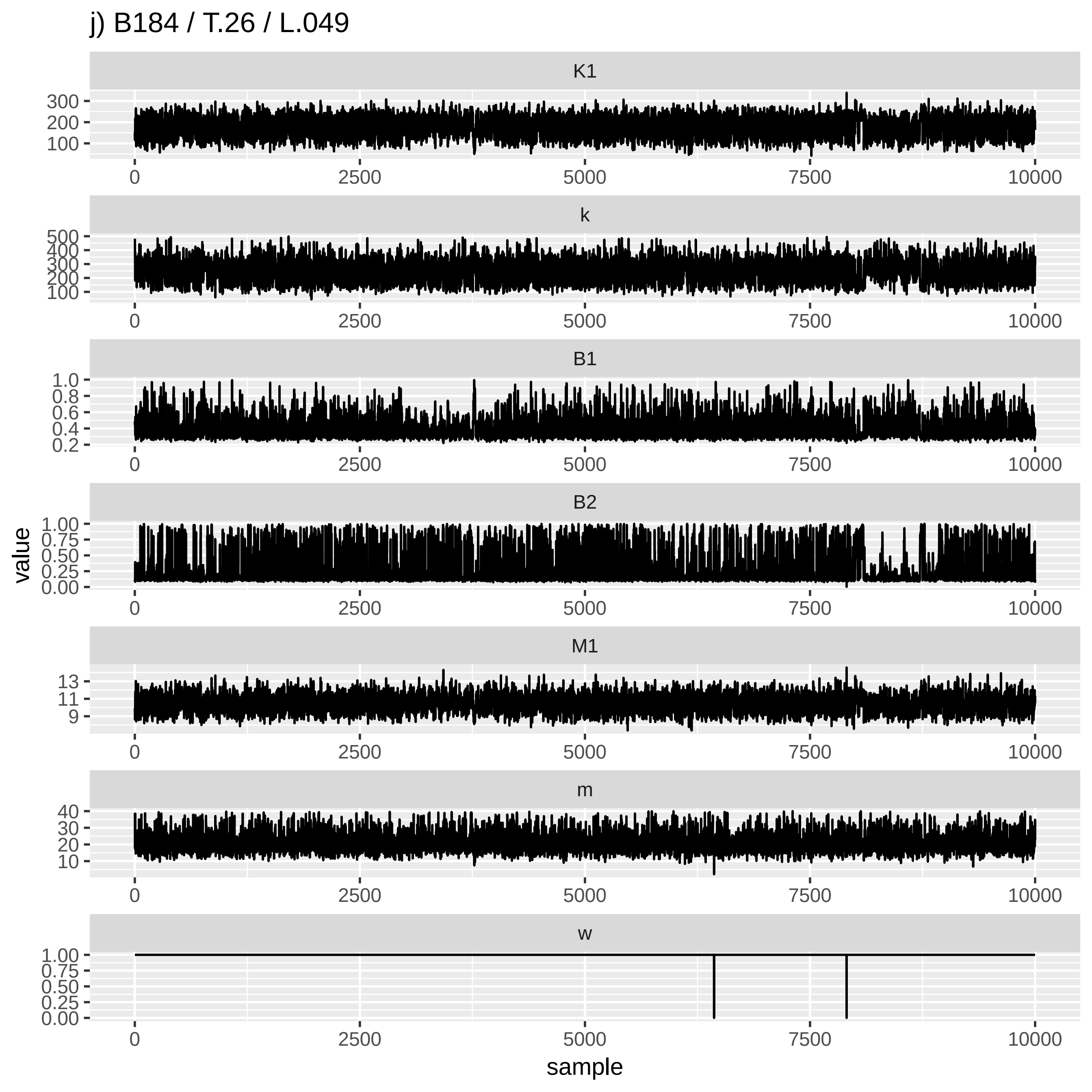
j) B184 / T.26 / L.049
K1
k
B1
B2
M1
m
w
value
sample
0
2500
5000
7500
10000

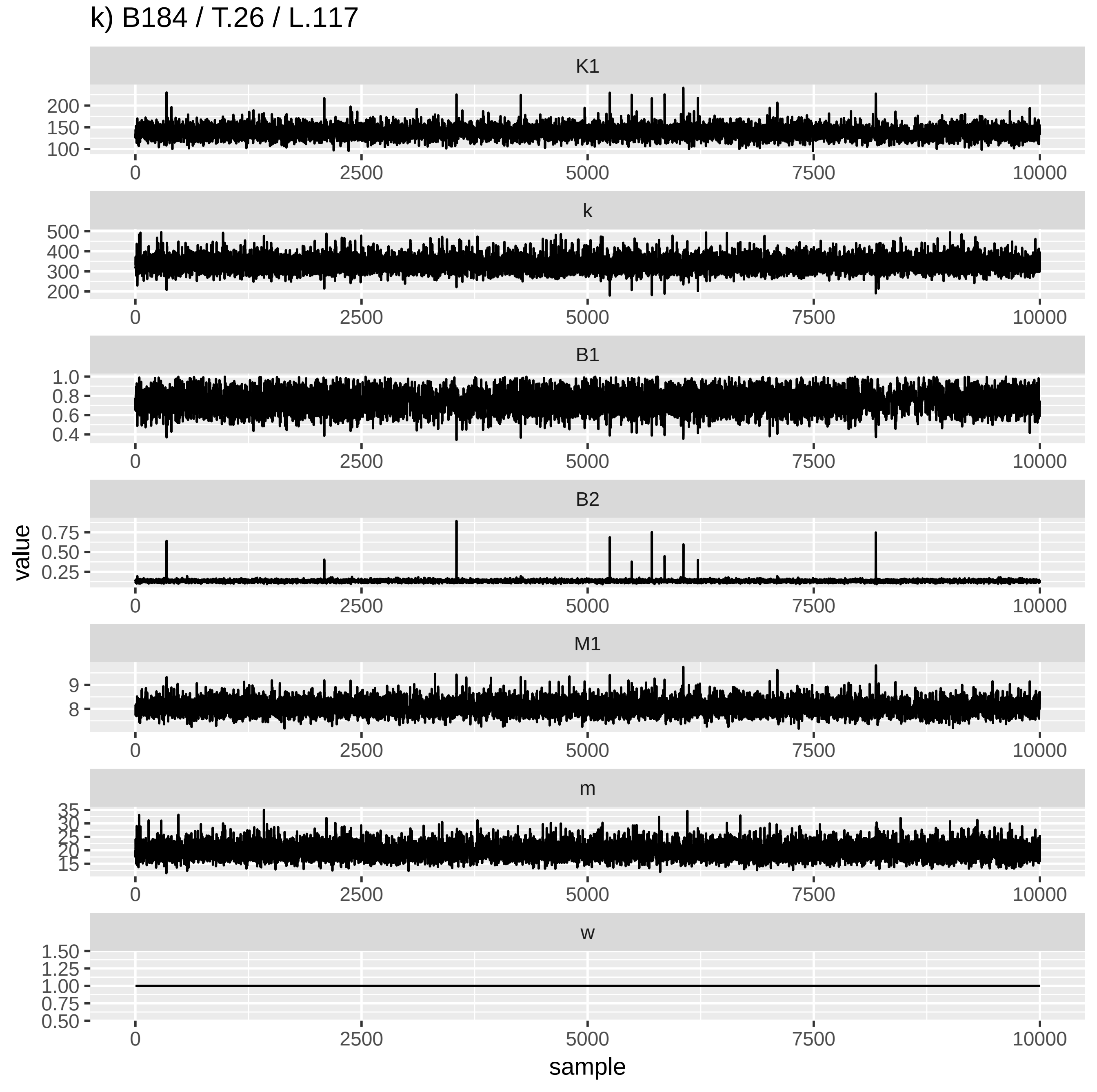
k) B184 / T.26 / L.117
K1
k
B1
B2
M1
m
w
value
sample
0
2500
5000
7500
10000

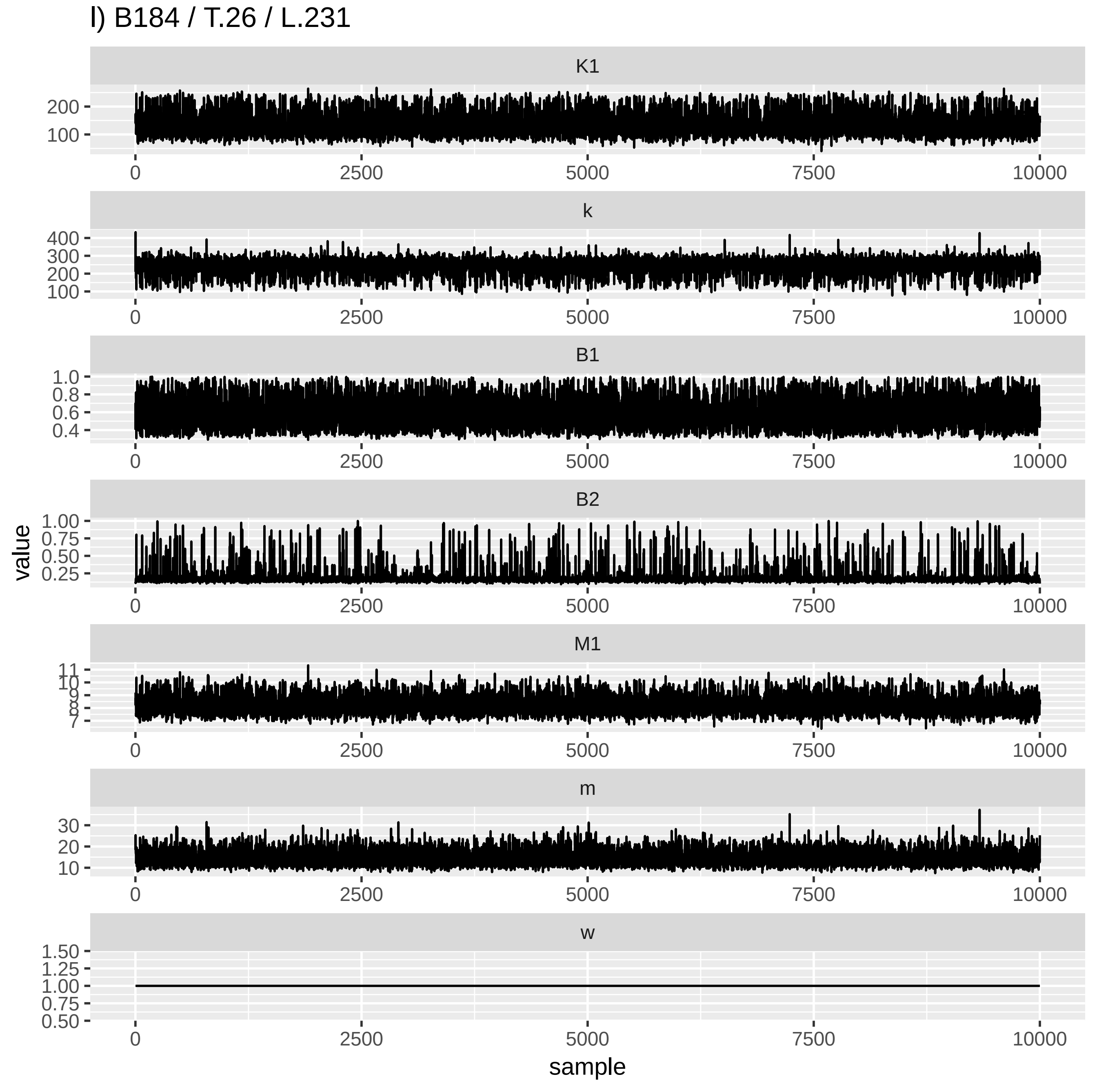
l) B184 / T.26 / L.231
K1
k
B1
B2
M1
m
w
value
sample
0
2500
5000
7500
10000

m) B224 / T.18 / L.049

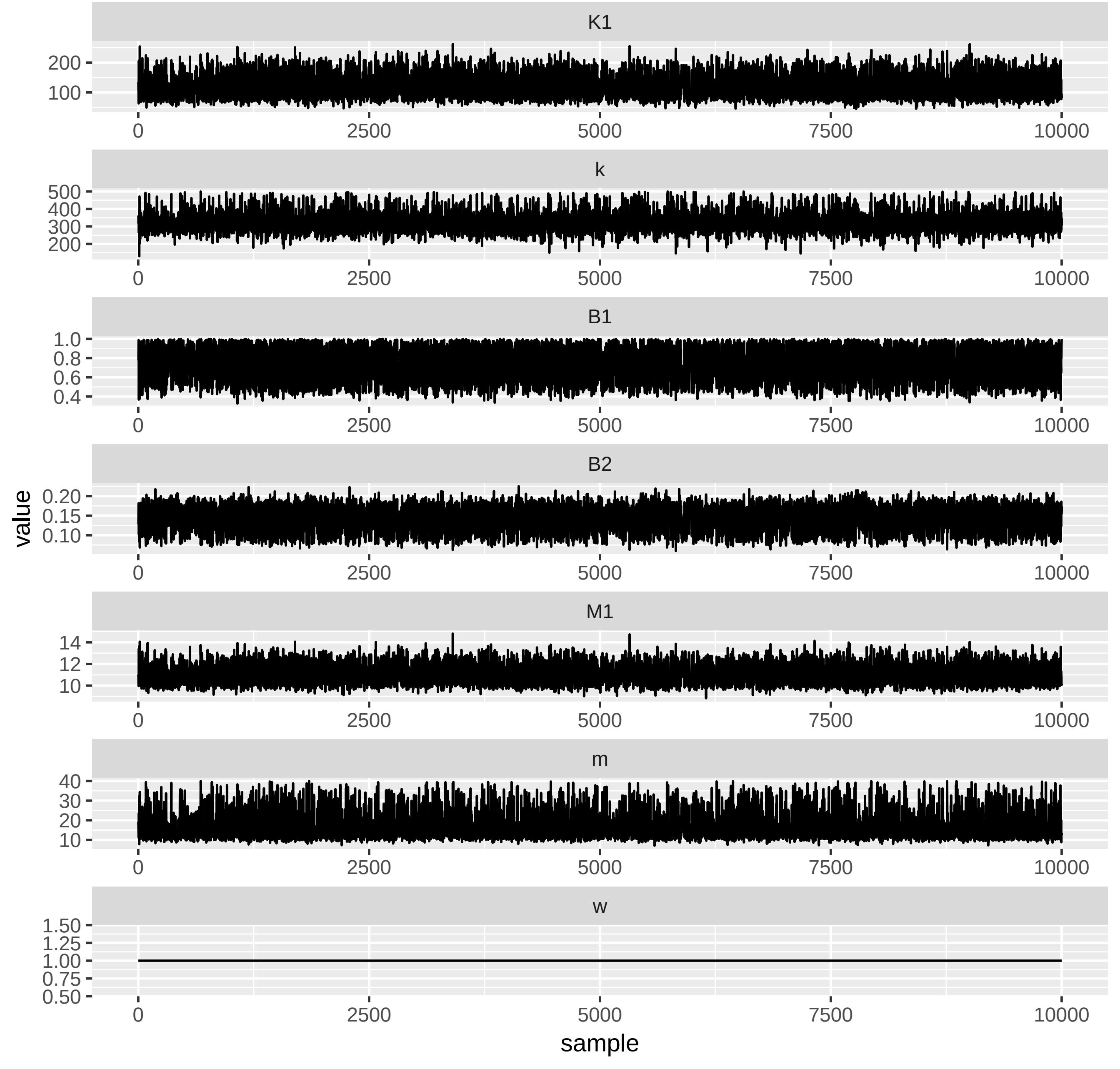

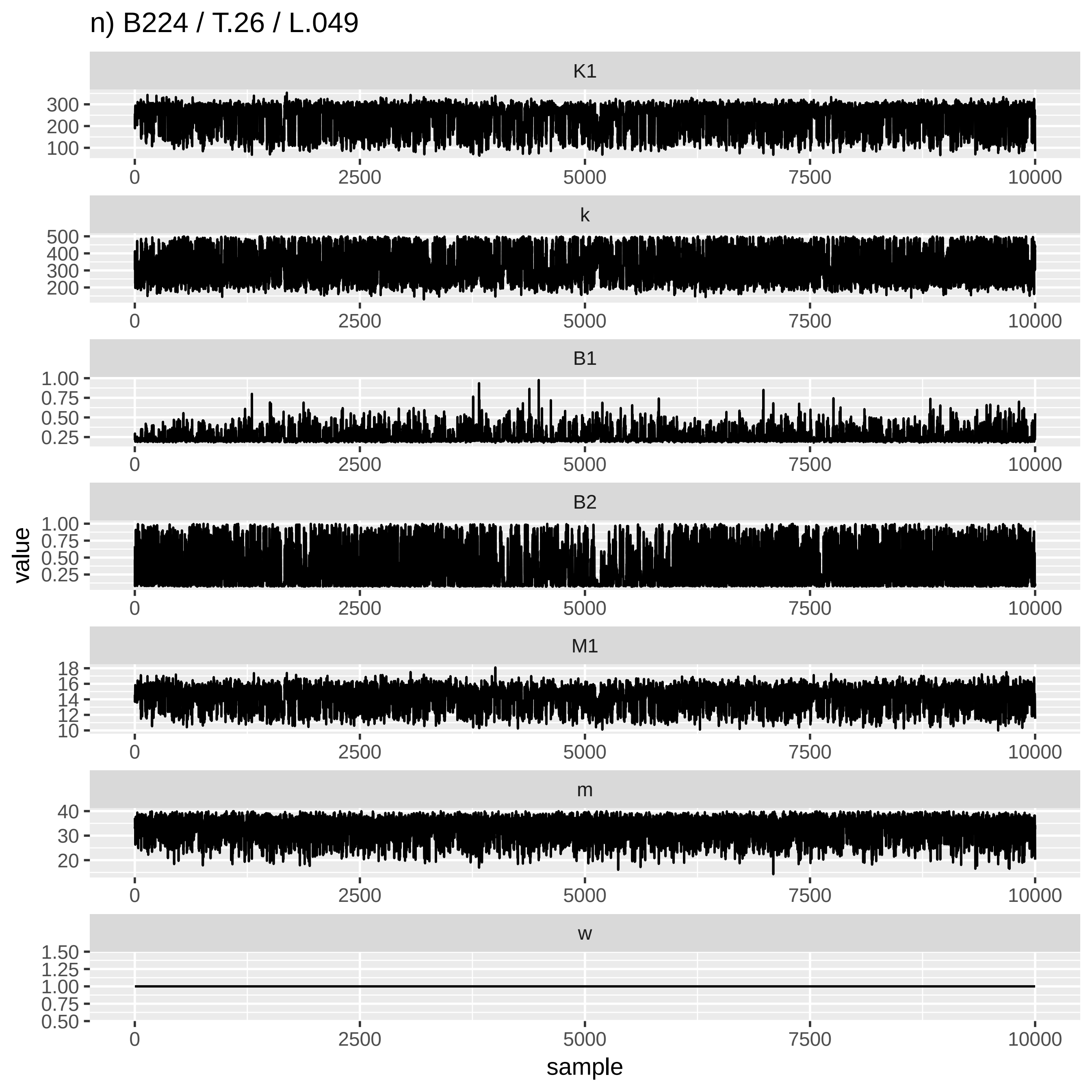

n) B224 / T.26 / L.049
K1
300
200
100
k
500
400
300
200
B1
1.00
0.75
0.50
0.25
B2
1.00
0.75
0.50
0.25
M1
18
16
14
12
10
m
40
30
20
w
1.50
1.25
1.00
0.75
0.50
0
2500
5000
7500
10000
value
sample

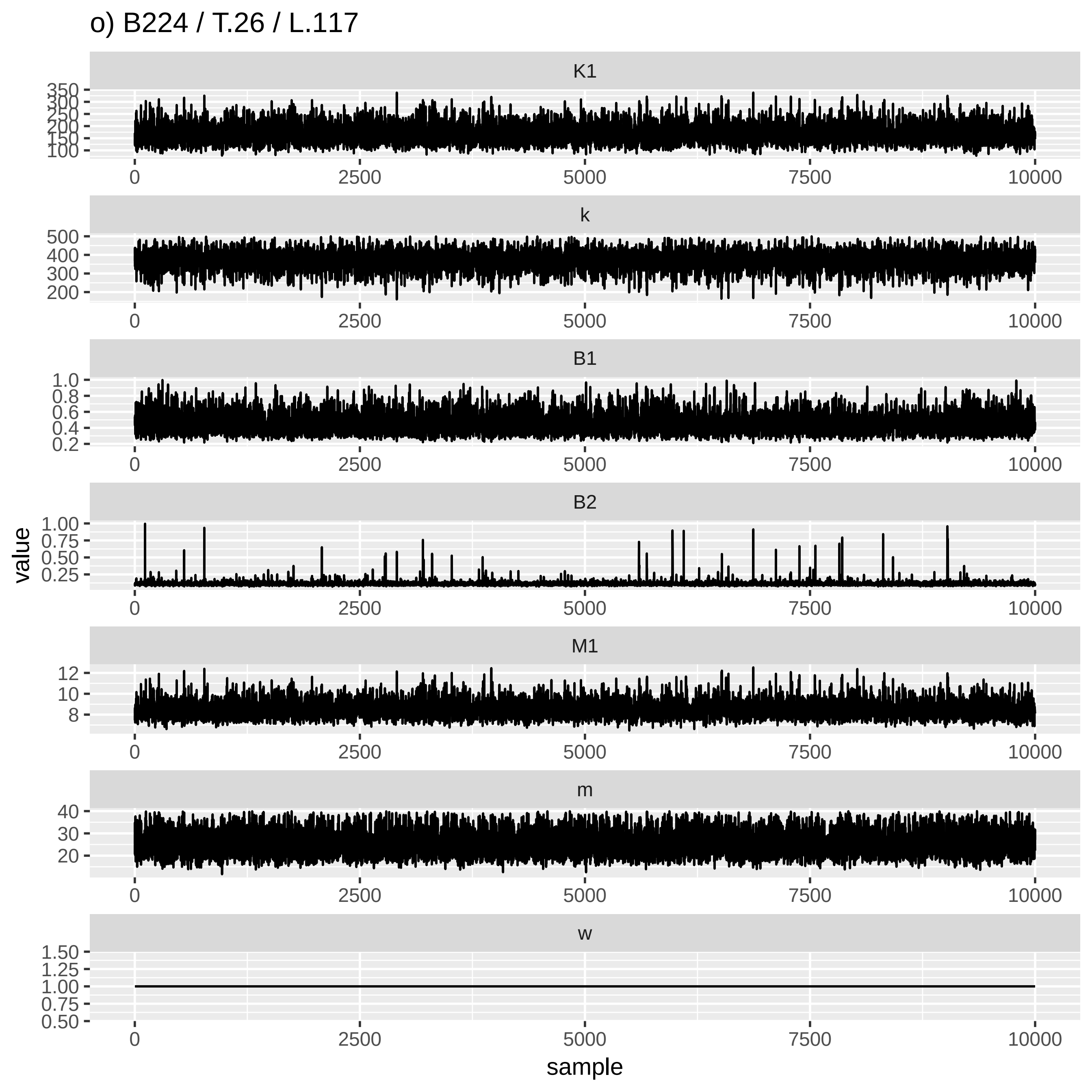

o) B224 / T.26 / L.117
K1
350
300
250
200
150
100
k
500
400
300
200
B1
1.0
0.8
0.6
0.4
0.2
B2
1.00
0.75
0.50
0.25
M1
12
10
8
m
40
30
20
w
1.50
1.25
1.00
0.75
0.50
0
2500
5000
7500
10000
value
sample

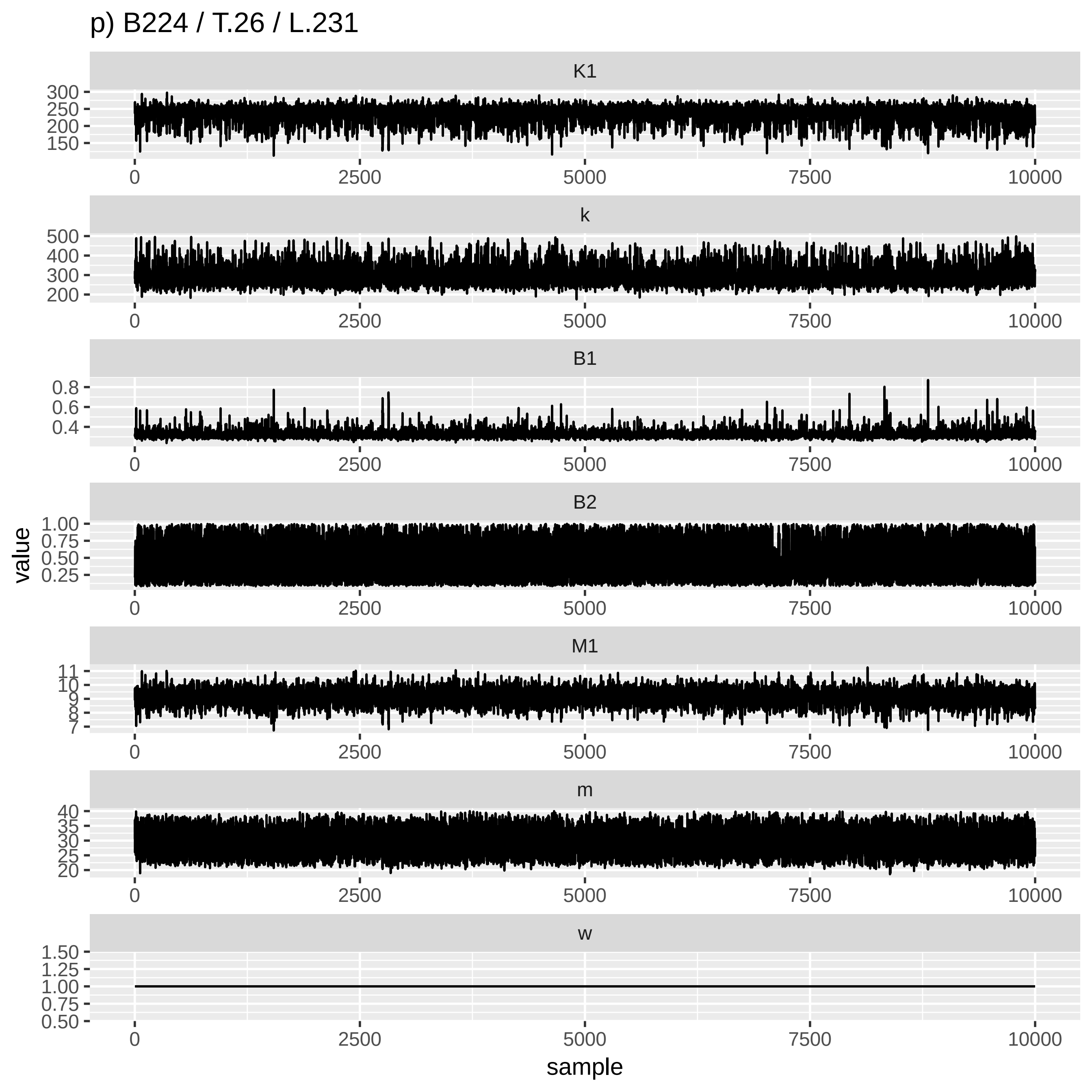

p) B224 / T.26 / L.231
K1
k
B1
B2
M1
m
w
value
sample
0
2500
5000
7500
10000

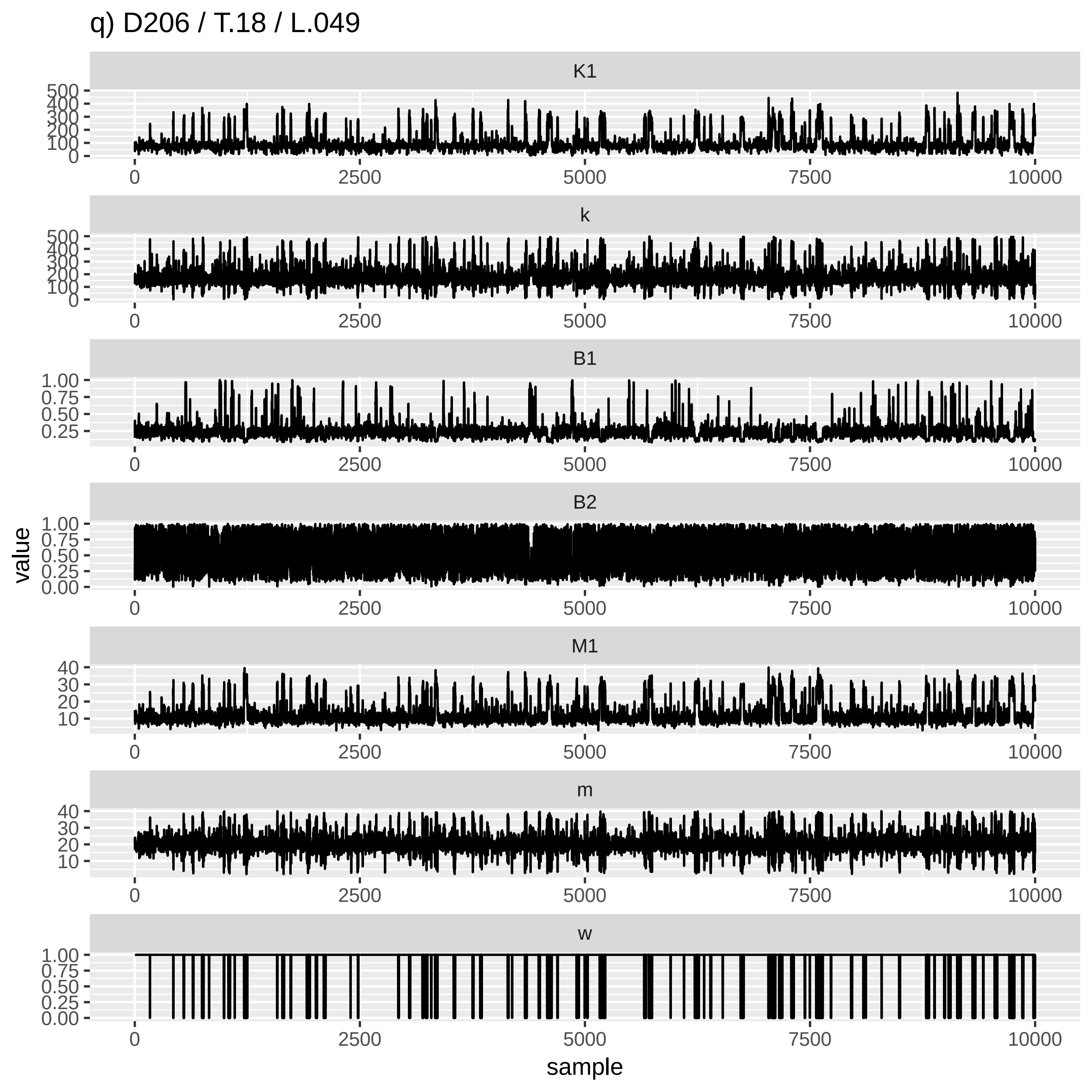

q) D206 / T.18 / L.049
K1
k
B1
B2
M1
m
w
value
sample
0
2500
5000
7500
10000

r) D206 / T.26 / L.049

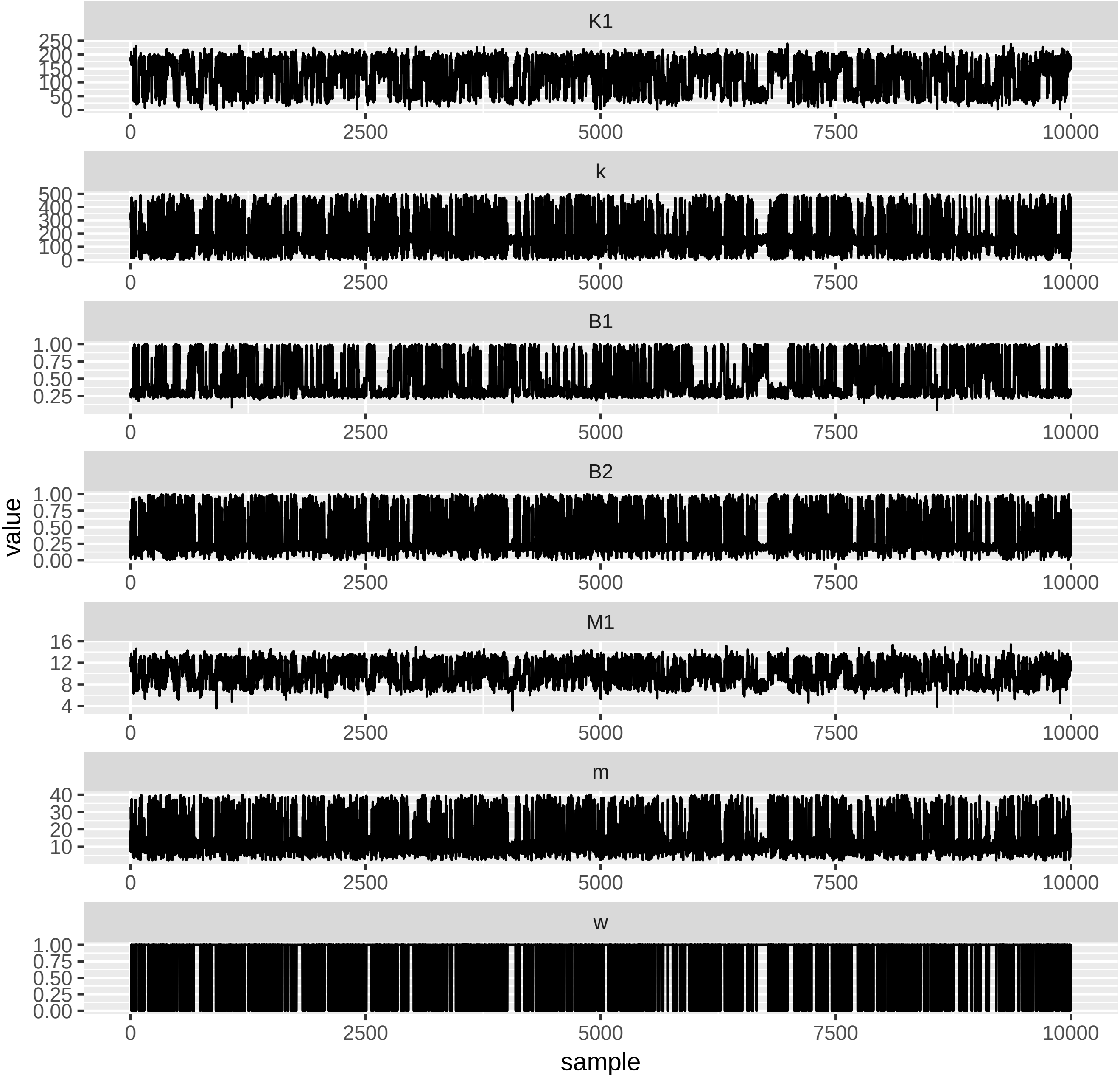

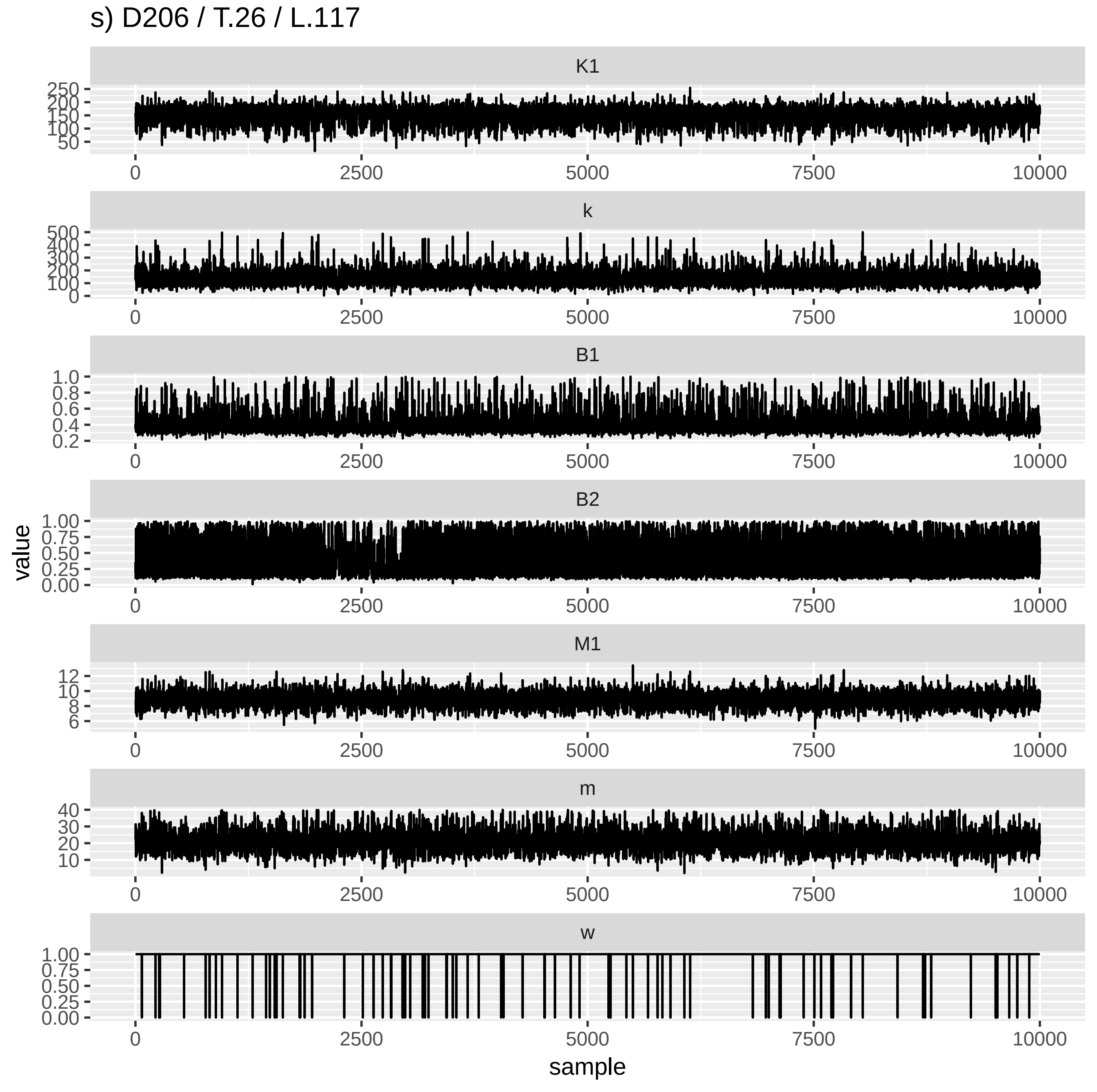

s) D206 / T.26 / L.117
K1
k
B1
B2
M1
m
w
value
sample
0
2500
5000
7500
10000

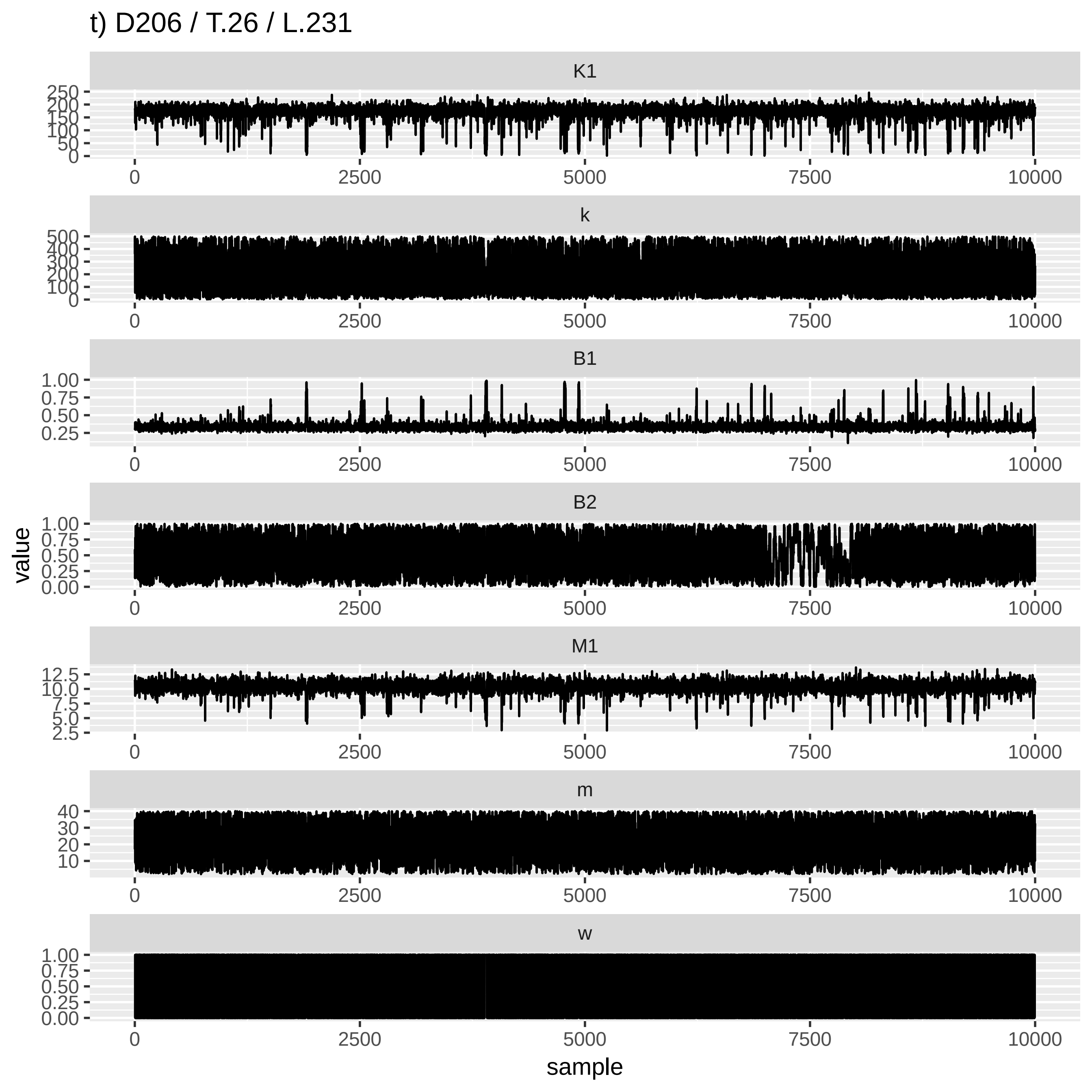
t) D206 / T.26 / L.231
K1
k
B1
B2
M1
m
w
value
sample
0
2500
5000
7500
10000
